# Enhancing NMR Signals in Liquids by Fluorine‐19 Overhauser Dynamic Nuclear Polarization (DNP) and Hyperpolarization Transfer to Carbon‐13

**DOI:** 10.1002/anie.202517498

**Published:** 2025-10-23

**Authors:** Maik Reinhard, Alex van der Ham, Luming Yang, Leonard Bröker, Tomas Orlando, Igor Tkach, Marcel Levien, Marina Bennati

**Affiliations:** ^1^ Electron‐Spin Resonance Spectroscopy Max Planck Institute for Multidisciplinary Sciences Am Fassberg 11 37077 Göttingen Germany; ^2^ Institute of Physical Chemistry University of Göttingen Tammannstr. 6 37077 Göttingen Germany; ^3^ Present address: Department of Chemical and Biological Physics Weizmann Institute of Science Herzl St 234 Rehovot 76100 Israel; ^4^ Present address: National High Magnetic Field Laboratory and Florida State University 1800 E. Paul Dirac, Dr. Tallahassee FL USA; ^5^ Present address: Institut des Sciences et Ingénierie Chimiques École Polytechnique Fédérale de Lausanne (EPFL) Lausanne CH‐1015 Switzerland

**Keywords:** DNP, Fluorine, Hyperpolarization, NMR, Overhauser

## Abstract

Nuclear magnetic resonance (NMR) plays an essential role in life and material science but suffers from low sensitivity due to the small energy differences it aims to detect. Therefore, techniques that can increase spin polarization, and thus sensitivity, in NMR are gaining tremendous attention. Overhauser effect dynamic nuclear polarization (OE‐DNP) can increase multidimensional NMR signals of small molecules in liquid solutions; however, at high magnetic fields, the direct mechanism to ^13^C is site selective, while it is poorly efficient for ^1^H. Here, we report OE‐DNP hyperpolarization of fluorine‐19 (^19^F) nuclei at 9.4 T, thus expanding the methodological toolbox of ^19^F NMR. Signal enhancements up to ∼20 under steady‐state conditions can be used to either rapidly detect ^19^F resonances or to enhance through‐bond *J‐*coupled ^13^C nuclei by two orders of magnitude. The latter corresponds to a 10^4^ gain in signal acquisition. We demonstrate this with ^19^F→^13^C INEPT (insensitive nuclei enhanced by polarization transfer) spectra of the drug flutrimazole at ^13^C natural abundance, where quaternary carbons become readily detectable, opening perspectives for accelerated NMR on fluorinated drugs or small molecules. The results are corroborated by a theoretical analysis of ^19^F OE‐DNP coupling factors.

## Graphical abstract

Fluorine nuclear spins can be hyperpolarized in solution by Overhauser dynamic nuclear polarization from a paramagnetic agent like galvinoxyl. This hyperpolarization can be transferred by nuclear magnetic resonance (NMR) to nearby ^13^C, leading to ^13^C signal enhancements up to ∼100. The method opens new opportunities for NMR structural determination of fluorine‐containing drugs and synthetic molecules.

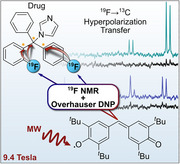

## Introduction

Nuclear magnetic resonance (NMR) spectroscopy is an indispensable technique to elucidate the structure of molecular systems with applications ranging from material sciences, physics, and biology to medicine. A nucleus of importance for NMR spectroscopy is fluorine‐19 (^19^F).^[^ ^1^, ^2^, ^3^, ^4^, ^5^, ^6^, ^7^, ^8^, ^9^ ^]^ It is one of the most sensitive NMR nuclei due to its large gyromagnetic ratio, (γ_19F_/γ_1H_ = 0.941), 100% natural abundance, and having a spin *I* = ½, typically yielding simple NMR spectra. ^19^F NMR finds applications in biomolecular NMR,^[^ ^1^, ^2^, ^3^, ^4^, ^10^, ^11^, ^12^ ^]^ material,^[^ ^13^, ^14^ ^]^ and environmental sciences.^[^ ^15^ ^]^ About 20% of commercial pharmaceuticals are fluorinated^[^ ^16^, ^17^ ^]^ and NMR spectroscopy has proven to be an important method for investigations of these drugs in solution^[^ ^6^, ^7^, ^8^, ^11^, ^18^, ^19^, ^20^, ^21^ ^]^ and solid state.^[^ ^4^, ^22^, ^23^, ^24^, ^25^, ^26^ ^]^ Yet, NMR suffers from an intrinsically low sensitivity stemming from the small population difference of the probed magnetic spin states.^[^ ^27^ ^]^ This problem can be alleviated by increasing the nuclear polarization above its thermal Boltzmann equilibrium, a process called hyperpolarization. Methods for sensitivity enhancement of ^19^F NMR were reported in the past via photochemically induced dynamic nuclear polarization (photo‐CIDNP),^[^ ^28^, ^29^, ^30^ ^]^ Overhauser effect dynamic nuclear polarization (OE‐DNP) in liquids at low magnetic fields^[^ ^31^, ^32^, ^33^, ^34^, ^35^, ^36^, ^37^ ^]^ up to 5 T,^[^ ^38^ ^]^ SABRE in benchtop NMR,^[^ ^39^ ^]^ and dissolution DNP (dDNP).^[^ ^40^, ^41^ ^]^ For example, dDNP was used in the context of general liquid‐state hyperpolarization to directly hyperpolarize metabolites^[^ ^42^, ^43^, ^44^ ^]^ or biomolecules via hyperpolarized water.^[^ ^44^, ^45^, ^46^, ^47^, ^48^ ^]^ It was also possible to measure the binding affinity of ligands with signal enhancements reaching several thousand for ^19^F.^[^ ^40^, ^41^ ^]^ On the other hand, as hyperpolarization in dDNP is generated ex situ and cannot be easily replenished, multidimensional NMR has so far only been reported with rapid acquisition techniques of limited resolution.^[^ ^44^, ^45^, ^47^, ^48^, ^49^ ^]^ Lastly, ^19^F signal enhancements were also reported recently in biomolecular solids using ^19^F MAS‐DNP^[^ ^50^, ^51^, ^52^ ^]^ even in biological cells,^[^ ^53^, ^54^ ^]^ which again highlights the interest of implementing hyperpolarization techniques to ^19^F NMR.

One approach to hyperpolarizing NMR signals in liquids is provided by OE‐DNP,^[^ ^55^, ^56^ ^]^ which transfers spin polarization from electron to nuclear spins through electron‐nuclear cross‐relaxation.^[^ ^57^, ^58^ ^]^ For this, a polarizing agent (PA), usually an organic radical, is mixed with the target solution of interest, and its EPR transitions are pumped by continuous wave (CW) microwave (MW) irradiation. OE‐DNP in liquids gained considerable attention when ^13^C signal enhancements of up to 1000 were observed at intermediate fields of 3.4 T. ^[^ ^59^ ^]^ Subsequent studies reported ^13^C signal enhancements up to 600 at 9.4 T and 60 at 14 T.^[^ ^60^, ^61^ ^]^ It was recognized that rapid molecular collisions on the picosecond and sub‐picosecond time scales, which are ubiquitous in liquids, can modulate the isotropic hyperfine coupling (hfc) *A*_iso_ and drive cross‐relaxation with rates of hundreds of GHz, i.e., on the order of the electron Larmor frequency at high (≳ 3 T) polarizing magnetic fields.^[^ ^59^, ^60^, ^62^, ^63^ ^]^ Further studies on the OE‐DNP polarization mechanism revealed that the chemical structure of the PA with respect to the target molecule plays a crucial role, because it largely affects complex formation and molecular dynamics.^[^ ^64^, ^65^, ^66^, ^67^, ^68^ ^]^ Complementary to this development, nuclei such as ^31^P^[^ ^38^, ^61^, ^64^, ^69^, ^70^, ^71^, ^72^, ^73^ ^]^ and ^1^H (also via solid‐state mechanism)^[^ ^74^, ^75^, ^76^, ^77^, ^78^ ^]^ have been explored in the context of liquid‐state DNP at high magnetic fields. Recently, we reported an NMR OE‐DNP instrument that enables MW irradiation of microliter sample volumes (∼20 µL) in liquids at 9.4 T.^[^ ^79^ ^]^ We showed that ^13^C NMR signal enhancements on the order of 2–100 can be obtained in many small organic molecules using nitroxide radicals as the PA,^[^ ^68^, ^80^ ^]^ and high‐resolution two‐dimensional (2D) NMR experiments with extensive signal averaging are compatible with OE‐DNP conditions.^[^ ^80^, ^81^ ^]^ This is the advantage of OE‐DNP over liquid‐state hyperpolarization techniques, in which hyperpolarization is performed ex situ and acquisition of high‐resolution multidimensional NMR remains challenging.

Although ^19^F is sometimes considered an isostere of ^1^H, it has significantly different electronic properties. It has been known since the early days of magnetic resonance that the fluorine atom is extremely sensitive to electronic spin densities at its nucleus, resulting in unusually large hfcs.^[^ ^82^, ^83^ ^]^ It has been observed experimentally by electron nuclear double resonance (ENDOR) spectroscopy that the hfc to ^19^F can be one order of magnitude larger than to ^1^H at the same chemical position.^[^ ^84^ ^]^ Thus, ^19^F appears particularly suited for OE‐DNP, which relies on large hfcs at the ^19^F nucleus. Particularly, Chandrakumar and George reported that the large chemical shift dispersion of ^19^F makes this nucleus attractive for NMR at intermediate fields (1–3 T) in combination with OE‐DNP.^[^ ^31^ ^]^ They also established the superior behavior of galvinoxyl PA with respect to TEMPO, leading to significant ^19^F signal enhancements up to 20 at about 3 T. Using our 9.4 T setup, we recently observed that these ^19^F NMR signal enhancements persist at high fields; however, the observation was not further investigated.^[^ ^80^ ^]^

Here, we demonstrate that ^19^F OE‐DNP at 9.4 T leads to sizable signal enhancements on ^19^F‐containing drug molecules and other small molecules, offering new opportunities for NMR structural investigation. In a first step, we report ^19^F OE‐DNP efficiency across a variety of functional groups. In a subsequent step, we employ ^19^F as a source of hyperpolarization, which can be transferred to other nuclei, specifically ^13^C, taking advantage of the larger gyromagnetic ratio γ19F/γ19Fγ13Cγ13C≈3.7, With this strategy, we can hyperpolarize quaternary carbons, which are key building blocks of organic molecules and so far, not amenable to any OE‐DNP scheme. We demonstrate the approach on the pharmaceutical drug flutrimazole with an overall sample amount of ∼2 µmol. Finally, we rationalize these findings by a consistent quantitative description for ^19^F OE‐DNP coupling factors, published in the literature over five decades, using semi‐classical relaxation theory.

## Results and Discussion

### Screening of ^19^F Overhauser Effect DNP on Various Small Molecules

**Figure 1:**
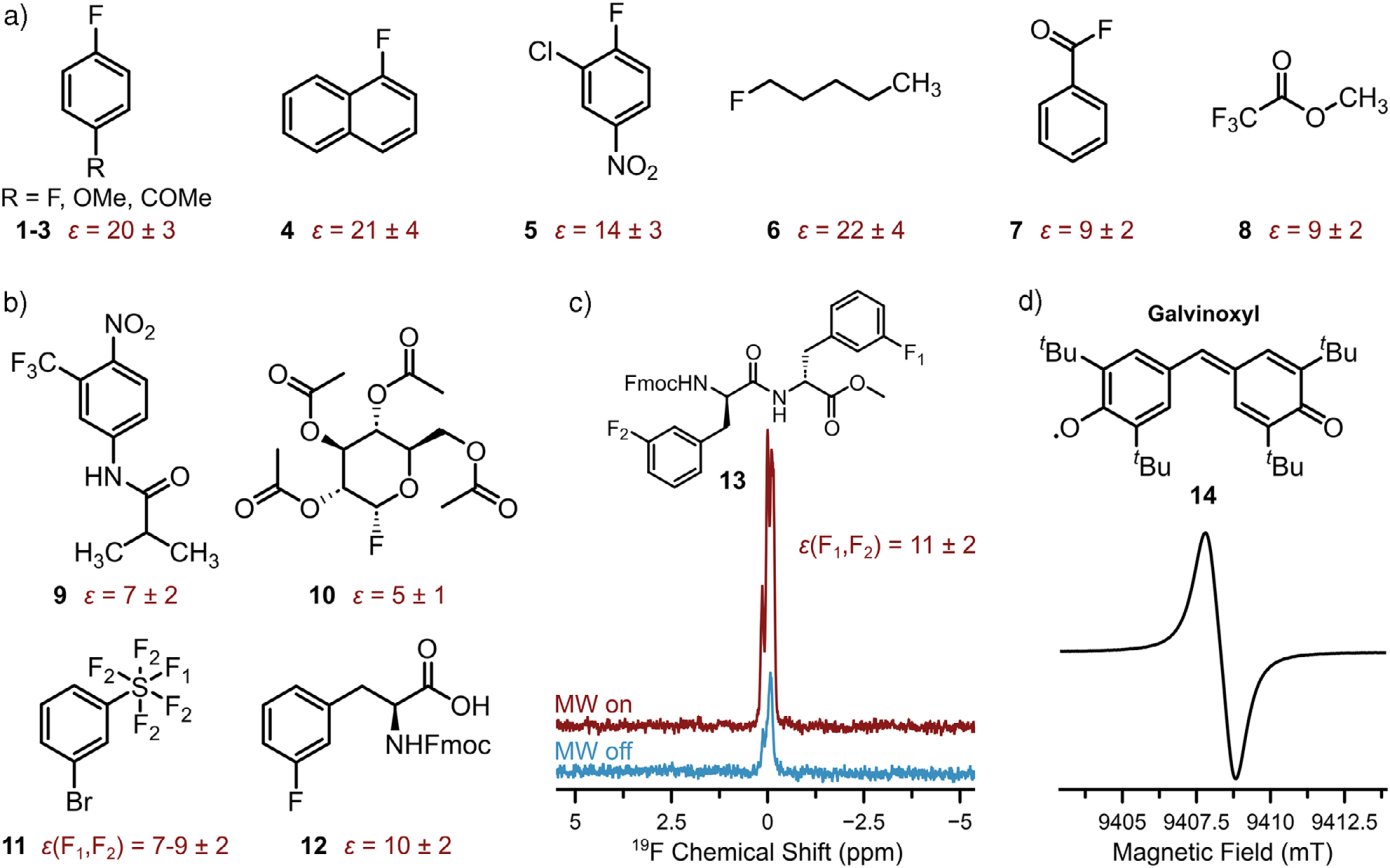
a) Molecular structure of small building block molecules and their ^19^F OE‐DNP enhancements at 9.4 T using ∼500 mM of target molecule in CCl_4_.1,4‐Difluorobenzene **1,** 4‐fluoroanisole **2,** 4‐fluoroacetophenone **3**, 1‐fluoronaphthalene **4**, 3‐chloro‐4‐fluoronitrobenzene **5**, 1‐fluoropentane **6**, benzoylfluoride **7**, methyl trifluoroacetate **8**. b) Molecular structure and ^19^F OE‐DNP signal enhancement (red) at 9.4 T of selected molecules. Flutamide **9** (∼10 mM in CCl_4_/DMSO, 500/12, v/v), 2,3,4,6‐tetra‐O‐acetyl‐α‐D‐glucopyranosyl fluoride **10** (∼400 mM in CHCl_3_), (3‐bromophenyl)sulfur pentafluoride **11** (∼500 mM in CCl_4_), and ʟ‐Fmoc‐3‐fluorophenylalanine **12** (∼2.5 mM in CCl_4_/CHCl_3_, 9/1, v/v). Data of **9** was published in Ref. [80] 1D spectra of **a** and **b** are reported in Figures S1 and S2. c) Structure of the dipeptide system ʟ‐Fmoc‐3‐fluorophenylalanyl‐ʟ‐3‐fluorophenylalanine methyl ester **13** (∼2.5 mM in CCl_4_/CHCl_3_, 9/1, v/v) and the corresponding MW on (red) and MW off (blue) ^19^F NMR spectra at 9.4 T. Considering the probe geometry,^[^ ^79^ ^]^ the amount of target molecules in the irradiated sample was ∼50 nmol. Spectra were scaled to the same noise level. The chemical shift scale is arbitrarily referenced to 0 ppm. ^19^F NMR/OE‐DNP experimental parameters: *V*_sample_ ≈ 20 µL, *t*_p_(90° RF) = 17 µs, *P*_RF_ = 21 W, recycle delay (RD) = 5 s, number of scans (ns, MW on) = 128, ns (MW off) = 1024. Processing parameters: LB (^19^F) = 3 Hz. All experiments were with a MW output power *P*_MW_ of ∼50 W (CW), at an effective sample temperature of ∼300 K and ∼10 mM galvinoxyl **14**. Experimental error on the enhancement is estimated to be about 15%. d) Liquid state 263 GHz CW EPR spectrum of ∼10 mM galvinoxyl in CCl_4_ at room temperature. Exp. EPR parameters: *ν*_MW_ = 263.185 GHz, *P*_MW_ = 0.5 mW, 100 kHz modulation frequency, 0.5 G modulation amplitude, ns = 1, 81.92 ms conversion time, *T* ≈ 300 K, *V*_sample_ ≈ 50 nL. Data shown in the figure were partially reported in the PhD thesis of one author.^[^ ^86^ ^]^

To explore the influence of the chemical environment on ^19^F NMR signal enhancements, we studied a variety of small molecules, in which the fluorine atom is part of various functional groups, using galvinoxyl as PA. For small aromatic building blocks, we observed ^19^F NMR signal enhancements up to ∼20, irrespective of other substituents on the ring (Figure 1a, **1**–**5**). For aliphatic compounds, the enhancements vary with ε ≈ 22 for 1‐fluoropentane, ε ≈ 9 for benzoylfluoride and methyl trifluoroacetate (Figure 1a, **6**, **7**, and **8**), and ε ≈ 8 −16 for *α*,*α*,*α*‐trifluorotoluene, diethyl fluoromalonate, and decafluoropentane reported earlier.^[^ ^80^ ^]^ These results suggest that fluorinated aromatic molecules are promising targets for ^19^F OE‐DNP in combination with galvinoxyl as PA. Accordingly, we selected a few representative molecules to showcase the potential applicability of ^19^F OE‐DNP. We report enhancements of ε ≈ 7 −9 for the drug flutamide **9** and (3‐bromophenyl)sulfur pentafluoride **11** in Figure 1b. The pentafluorosulfonyl group of **11** has recently gained interest in organic synthesis, as it acts as a strong electron‐withdrawing group (EWG) while being lipophilic.^[^ ^85^ ^]^ Similarly, glucose derivative **10** and ʟ‐Fmoc‐3‐fluorophenylalanine **12** in Figure 1b show promising enhancements up to ε ≈ 10. Interestingly, the signal enhancement of **12** is similar to the Me‐(3‐FPhe)_2_‐Fmoc dipeptide derivative **13** reported in Figure 1c, where we observe an average signal enhancement of ε ≈ 11. All individual spectra are reported in Section S2. We emphasize that signal enhancements of about one order of magnitude correspond to two orders of magnitude reduction in experimental time from spectral averaging. To explore the detection limit of ^19^F OE‐DNP in this setup, we measured 2 nmol of **12** and 5 nmol of fluorobenzene **15**, obtaining a signal to noise ratio (SNR) of 3.5 and 35, respectively, within 30–60 min of acquisition (Figures S2d and S3d). We note that we observed in some cases inhomogeneous line broadening (LB) of the signal up to ∼100 Hz, caused by the MW‐induced temperature gradient across the sample. However, this might be attenuated in the future by optimizing the sample cooling setup.

### ^19^F→^13^C Hyperpolarization Transfer

**Figure 2:**
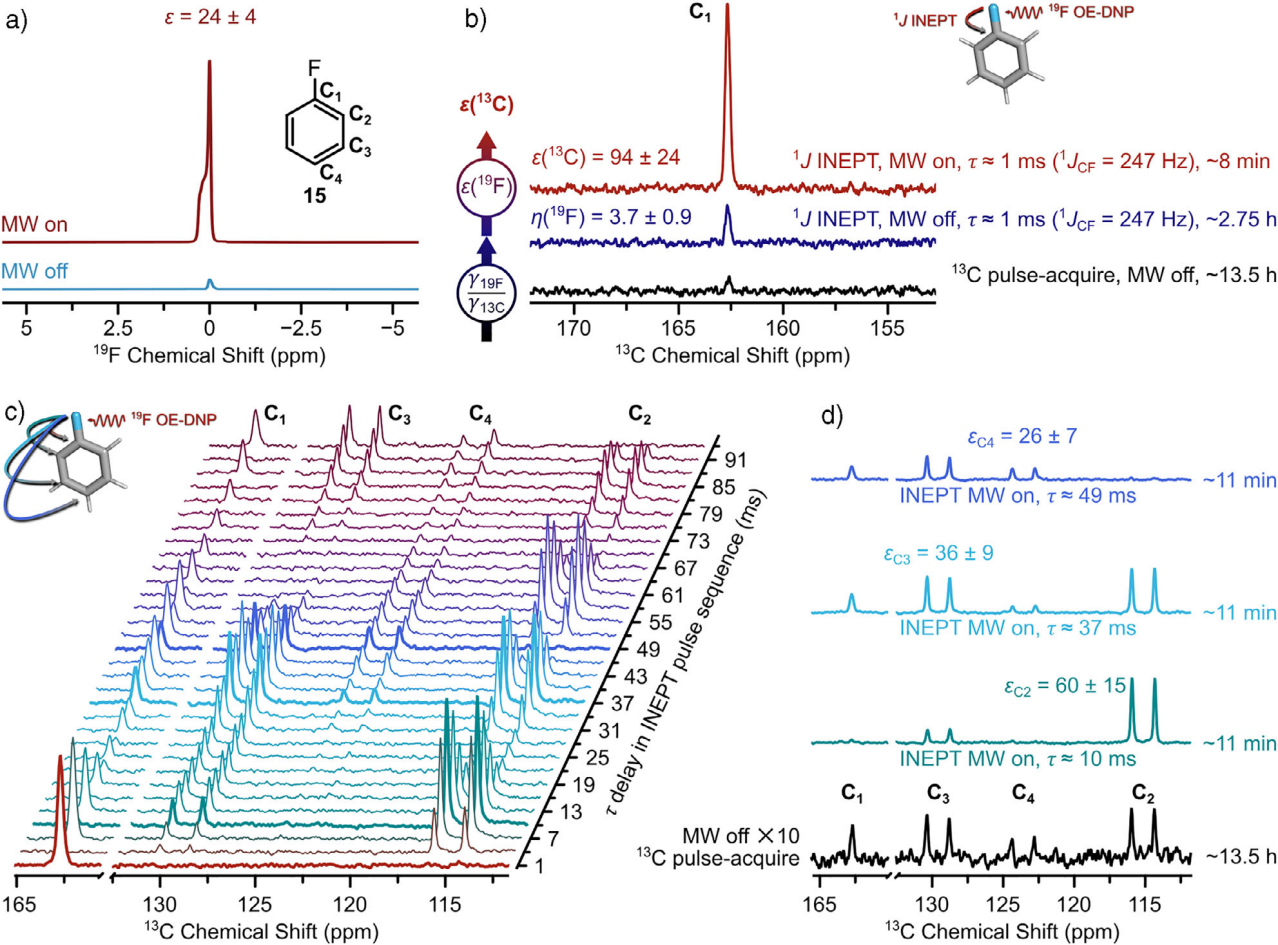
a) ^19^F OE‐DNP spectra of fluorobenzene (∼500 mM, natural abundance) in CCl_4_ doped with ∼10 mM galvinoxyl with (red) and without (blue) MW irradiation. Inset: chemical structure of fluorobenzene **15** with numbering of carbon atoms. b) C_1_ region of the ^13^C spectrum of fluorobenzene. Black spectrum is a pulse‐acquire experiment without MW irradiation; blue and red are ^19^F→^13^C INEPT experiments without and with MW irradiation, respectively. Vertical arrows indicate enhancements comparing the different experiments (*η* = *γ*_19F_/*γ*_13C_ and *ε*(^19^F)) with an error estimated to be 25%, due to the poor signal‐to‐noise ratio of the ^13^C Boltzmann spectrum (black). c) Pseudo‐2D plot of the ^19^F→^13^C OE‐DNP/INEPT spectra of **15**, where the inter‐pulse delay *τ* in the INEPT sequence is systematically incremented. d) Slices of the ^19^F→^13^C OE‐DNP/INEPT spectra, marked in bold in (c), corresponding approx. to *τ* = 1/(4 *^n^J*_C,F_), with *^n^J*_C,F_ (*n* = 2–4). The black spectrum is the thermal ^13^C spectrum depicted in 2b. Notably, the black thermal spectrum and spectra from the pseudo‐2D plot were measured on two different samples of identical composition and under the same experimental conditions. C_2_–C_4_ resonances are split by ^1^ *J*_C,H_ couplings. Enhancements are obtained from comparison of the ^13^C pulse‐acquire spectrum (black) with an uncertainty of 25%. ^19^F NMR/OE‐DNP exp. parameters: *T* ≈ 300 K, *V*_sample_ ≈ 20 µL, *P*_MW_ ≈ 50 W (CW), *t*_p_(90° RF) = 17 µs, *P*_RF_ = 21 W, RD = 7 s, ns(MW on, MW off) = 4, exp. time: ∼25 s. Processing parameters: LB(^19^F) = 3 Hz. ^19^F→^13^C OE‐DNP/INEPT exp. parameters: *P*_MW_ ≈ 50 W (CW), *t*_p_(90° RF,C) = 10.5 µs, *t*_p_(90° RF,F) = 17 µs, *τ*_n_ = 1 (shown in b), 10, 37, 49 ms (shown in d), *P*_RF,C_ = 41 W, *P*_RF,F_ = 21 W, *P*_RF,F_ (decoupling) = 1.2 W, RD (INEPT) = 6 s, RD (pulse‐acquire) = 30 s, ns (OE‐DNP/INEPT) = 64, ns (INEPT, MW off) = 1280, ns (pulse–acquire, MW off) = 1536, ns (pseudo‐2D OE‐DNP/INEPT) = 96. All spectra were recorded with broadband ^19^F decoupling using GARP4^[^ ^90^ ^]^ composite pulse decoupling (CPD). Exp. time: ∼8–11 min for OE‐DNP/INEPT, ∼2.75 h for INEPT (no MW), ∼13.5 h for pulse‐acquire, ∼6 h for the pseudo‐2D exp in (c). Processing parameters: LB (^13^C) = 10 Hz. Data shown in the figure were partially reported in the PhD thesis of one author.^[^ ^86^ ^].^

Besides direct OE‐DNP and detection of ^19^F, we exploited the high ^19^F enhancements on aromatic molecules and the large gyromagnetic ratio of ^19^F to transfer spin polarization to adjacent ^13^C nuclei, which are crucial for structure determination but much less abundant (1.1%) as well as much less sensitive (with gyromagnetic ratios γ19F/γ19Fγ13Cγ13C≈3.7). Polarization transfer experiments in combination with OE‐DNP were previously reported at high fields using either ^13^C→^1^H reversed INEPT (insensitive nuclei enhanced by polarization transfer) or ^13^C‐^−13^C TOCSY (total correlation spectroscopy) and INADEQUATE (incredible natural abundance double quantum transfer experiment) pulse sequences.^[^ ^80^, ^87^, ^88^ ^]^ Here, we demonstrate that ^19^F enhancements can be exploited in ^19^F→^13^C INEPT experiments, where the ^19^F hyperpolarization and INEPT sensitivity multiplicatively contribute to increase the detected ^13^C signal. Figure 2a,b, illustrates the ^19^F OE‐DNP enhancement and a representative ^1^ *J* INEPT experiment (pulse sequence in Section S3) on fluorobenzene, in which the ^19^F polarization is transferred to the C_1_ carbon (natural abundance). The standard ^19^F→^13^C INEPT experiment is well‐known in conventional NMR^[^ ^89^ ^]^ and provides an enhanced ^13^C signal by a factor of about *η*(^19^F) = 3.7 ± 0.9 (Figure 2b). When MW is switched on, the additional OE‐DNP enhancement on ^19^F of about 24 is gained multiplicatively, leading to a signal enhancement of ε_1_ = 94 ± 24 as compared to ^13^C pulse‐acquire on the C_1_. Moreover, the polarization transfer experiments utilized a shorter recycle delay, exploiting the shorter *T*_1n_ of the fluorine source nucleus as compared to ^13^C, decreasing the experimental time further. Importantly, the LB observed in the ^19^F spectra under OE‐DNP are not reflected in the ^13^C spectra due to lower sensitivity of ^13^C chemical shifts to temperature (^13^C line widths: MW off ∼7–12 Hz, MW on (INEPT) ∼7–17 Hz, MW on (pulse‐acquire) ∼5–12 Hz).

Polarization transfer via the INEPT mechanism, which utilizes scalar *^n^J*_CF_ couplings, is not limited to spin pairs coupled via a single chemical bond (^1^ *J* coupling). Thus, in a second step, we explored transfer via ^2^ *J*, ^3^ *J*, and ^4^ *J* couplings. Here, the limitation is expected to be the smaller coupling size, which requires longer evolution times, on the order of 1/(4 *^n^J*_C,F_), and thus signal decay due to *T*_2_ *_n_* relaxation. Figure 2c illustrates a pseudo‐2D plot of ^19^F→^13^C OE‐DNP enhanced INEPT (abbreviated in the following as OE‐DNP/INEPT) showing the polarization transfer to C_2_, C_3_, and C_4_ as a function of the evolution time *τ* in the pulse sequence. A contour plot is displayed in Figure S8. The plot demonstrates the expected oscillation of the ^13^C intensities, which is not affected by the MW irradiation under OE‐DNP conditions. ^19^F decoupling was applied during acquisition, and the visible splitting of the ^13^C resonances is due to ^1^ *J*_C,H_ couplings. Figure 2d shows slices from the pseudo‐2D plot at evolution times that maximize the polarization transfer to C_2_–C_4_. As depicted in Figure 2d, C_2_ is enhanced by a factor of ε_2_ = 60 ± 15 via ^2^ *J* OE‐DNP/INEPT as compared to direct ^13^C detection. We note that the hyperpolarization is maintained throughout the INEPT sequence, as it only decays with *T*_1_ *_n_* of the ^19^F nucleus, and the limiting factor is *T*_2_ *_n_* relaxation. Moreover, all OE‐DNP/INEPT spectra were recorded with only 8–11 min acquisition time, as compared to the ∼13.5 h for the reference ^13^C pulse acquired MW off spectrum, where the signals are still very weak. This highlights the potential of this method to access structural investigation of compounds at low concentrations.

We also compared a two‐bond ^19^F→^13^C OE‐DNP enhanced INEPT with a one‐bond ^1^H→^13^C INEPT Boltzmann experiment, which yields a gain in enhancement as compared to ^1^H→^13^C INEPT of *κ* ≈ 15 (*κ* = *ε*(^2^ *J*_C,F_ INEPT DNP)∕*η*(^1^ *J*_C,H_ INEPT Boltzmann), Figure S7a). Here, the ^1^ *J*_C,H_ INEPT over the ^13^C{^1^H} pulse‐acquire was *η*(^1^H) ≈ 4.0 ± 0.3. Similarly, C_3_ and C_4_ are enhanced by a factor of ε_3_ = 36 ± 9 and ε_4_ = 26 ± 7 in the ^3^ *J* and ^4^ *J* optimized OE‐DNP/INEPT experiments, respectively. We note that these enhancements are larger than the direct OE‐DNP effect to ^13^C (ε = 6 −8), which we recently reported in Ref. [80] using nitroxides as PA and using galvinoxyl ε = 5 −7 (Figure S7b).

### Enhancing Signals of Quaternary Carbons on a Drug

**Figure 3:**
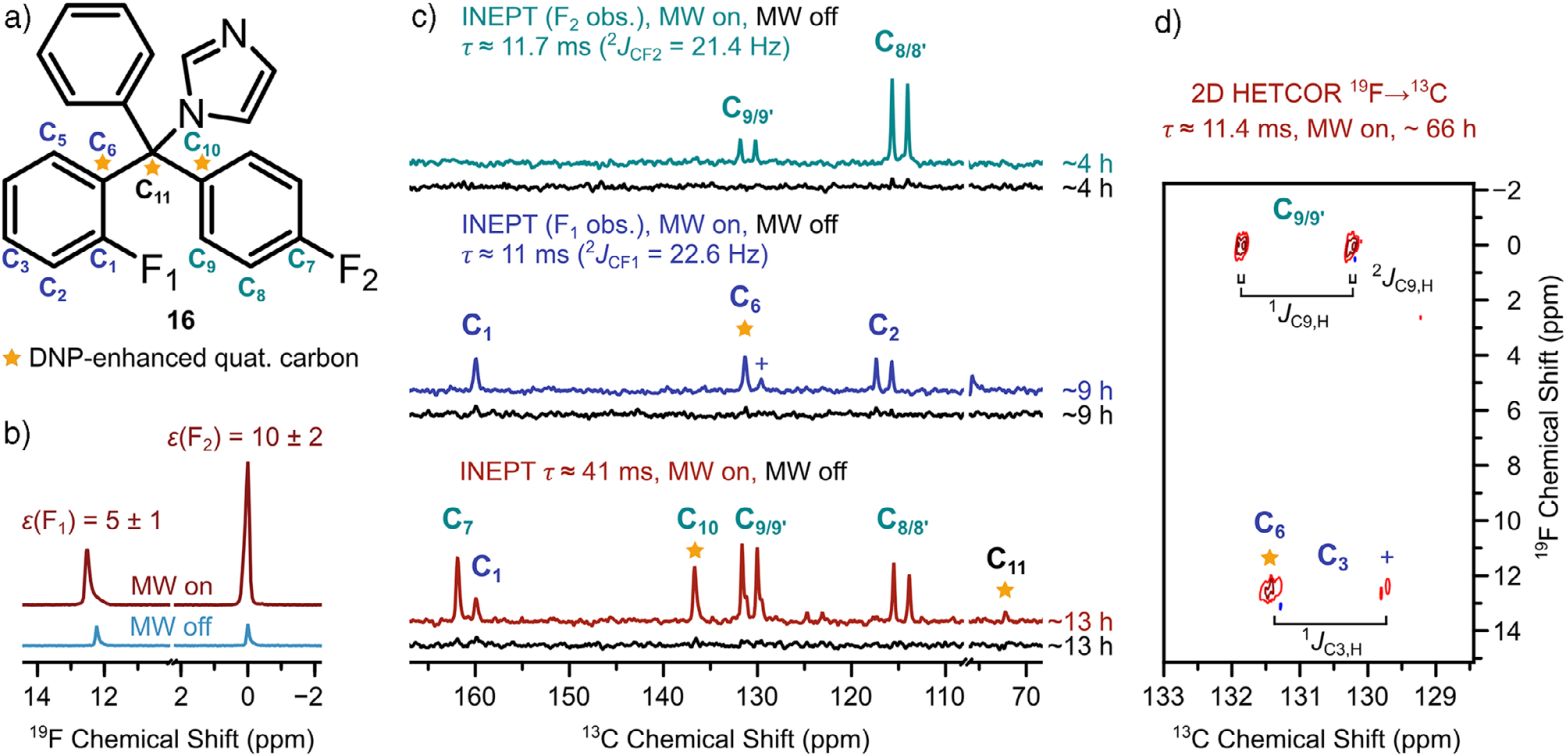
a) Chemical structure of flutrimazole **16** with numbering of fluorine and carbon atoms. Stars denote observed quaternary carbons. b) ^19^F OE‐DNP spectra of **16** (∼100 mM) in CCl_4_/CHCl_3_, 9/1, v/v doped with ∼15 mM galvinoxyl with (red) and without (blue) MW irradiation. c) ^19^F→^13^C OE‐DNP/INEPT spectra, *τ* = 1/(4 ^2^ *J*_C, F_), selective ^19^F pre‐saturation of F_1_ (green, *τ* = 11.7 ms F_2_ obs.) and F_2_ (blue, *τ* = 11 ms F_1_ obs.); ^19^F→^13^C OE‐DNP/INEPT spectrum (red) with no selective saturation and with *τ* = 41 ms; ^19^F→^13^C INEPT reference spectra without MW irradiation (black). The signal marked with (+) corresponds to one line of the doublet of C_3_, overlapping with the singlet of C_6_. d) 2D HETCOR spectrum under MW irradiation with *τ* = 11.4 ms. All spectra were recorded with broadband ^19^F decoupling using GARP4 CPD. NMR/OE‐DNP experimental parameters (pulse‐acquire, selective pre‐saturation INEPT, INEPT, and HETCOR pulse sequences): *T* ≈ 300 K, *V*_sample_ ≈ 20 µL, *P*_MW_ ≈ 50 W (CW), *t*_p_(90° RF, C) = 10.5 µs, *t*_p_(90° RF, F) = 17 µs, *t*_p_(RF, F, sat) = 0.5 s, *P*_RF, C_ = 41 W, *P*_RF, F_ = 21 W, *P*_RF, F, sat, DNP_ = 0.01 W, *P*_RF, F, sat, Boltz_ = 0.03 W, *P*_RF, F, dec_ = 1.2 W, RD = 2.5 s, RD(HETCOR) = 2 s, ns(^19^F, MW on, MW off) = 16, ns(INEPT (F_1_), MW on, MW off) = 8192, ns(INEPT (F_2_), MW on, MW off) = 4096, ns(INEPT, red, MW on, MW off) = 13 312, ns(HETCOR, MW on) = 2280, size of FID 512 × 32. Exp. time: ∼1 min for ^19^F NMR in (b), ∼9 h for OE‐DNP/INEPT F_1_ obs. (MW on and MW off), ∼4 h for OE‐DNP/INEPT F_2_ obs. (MW on and MW off), ∼13 h for non‐selective OE‐DNP/INEPT (red, MW on and MW off), and ∼66 h for 2D HETCOR. Processing parameters: LB (^19^F) = 3 Hz, LB (^13^C) = 20 Hz. For HETCOR: LB (^19^F, HETCOR) = 0.5 Hz, LB (^13^C, HETCOR) = 2 Hz, linear forward prediction parameters: number of raw data points to be used for processing (TDeff) = 32, number of linear prediction coefficients (NCOEF) = 2, number of points for linear prediction (LPBIN) = 512 in f1.

Fluorine is frequently incorporated into drug molecules,^[^ ^16^ ^]^ and thus ^19^F OE‐DNP offers new opportunities for their structural investigation. To demonstrate the application potential, we report in Figure 3b the ^19^F OE‐DNP spectrum of the commercial antifungal drug flutrimazole (Figure 3a). It contains two fluorine atoms, one on an ortho position (F_1_), the other on a para position (F_2_), with respect to the central quaternary carbon. These were observed to give ^19^F NMR enhancements of about 5 and 10, respectively. To ensure optimized excitation, simplify the assignment, and maximize spectral resolution, ^19^F→^13^C INEPT experiments were performed, in which one of the two ^19^F resonances was selectively saturated. Details on the pulse sequence are given in Figure S5. Figure 3c shows ^2^ *J* optimized ^19^F→^13^C OE‐DNP/INEPT spectra with selective pre‐saturation and on a sample of ∼2 µmol unlabeled drug. Similar to the model system fluorobenzene, we observed efficient ^19^F→^13^C hyperpolarization transfer via ^2^ *J* INEPT to C_2_, C_6_, and C_8/8′_ (Figure 3a,c). Notably, while these OE‐DNP/INEPT spectra were obtained within ∼4 and ∼9 h, no signals could be detected in the INEPT without MW irradiation at the same acquisition time (Figure 3c, MW off, black). A further comparison with a ^13^C pulse‐acquire experiment showed that no ^13^C signals were detectable (except the solvent) in the same sample even after 51 h of acquisition. Only when increasing the sample amount by a factor of 25 (∼50 µmol), peaks became observable after 17 h of acquisition, with comparable SNR as in the ^19^F→^13^C OE‐DNP/INEPT (Figure S11). Indeed, assuming an INEPT polarization transfer efficiency of about 64%, as observed for fluorobenzene ^2^ *J* OE‐DNP/INEPT, Figure 2d, ^19^F enhancements of 5–10 translate to ^13^C enhancements on the order of 12–24.

Furthermore, we also observe signals coming from ^1^ *J*_C,F_ and ^3^ *J*_C,F_ couplings (C_1_, C_3_, and C_9/9′_). Contrary to the model system fluorobenzene, flutrimazole contains quaternary carbons, a chemical motif that otherwise cannot be hyperpolarized by OE‐DNP, as they are lacking accessibility and directing groups for the PA. Moreover, quaternary carbons usually have longer *T*_1_ *_n_* and are not amenable to polarization transfer experiments such as ^1^ *J*_C,H/F_ INEPT.^[^ ^80^ ^]^ Specifically, the C_6_, C_10_, and C_11_ carbons are not bound to any ^1^H, and therefore cannot benefit from ^1^H→^13^C ^1^ *J*_C,H_ INEPT experiments. We were able to detect all these resonances using the ^19^F→^13^C OE‐DNP/INEPT experiment (Figure 3c blue and red, and Figure S11). In terms of resolution, we obtained linewidths of ∼3–20 Hz in ^13^C‐detected INEPT experiments.

As shown in Figure 3c, separation of the resonances from C_6_, C_3_, and C_9/9′_ is possible when using selective excitation of one of the ^19^F nuclei. Alternatively, to increase the resolution of the non‐selective OE‐DNP/INEPT experiment (red trace in Figure 3c), we have performed a 2D heteronuclear correlation (HETCOR) ^19^F→^13^C experiment (pulse sequence in Figure S5c), which is illustrated in Figure 3d for the C_6_/C_9/9′_ region. Through the additional chemical shift resolution in the ^19^F dimension, carbon doublets become well resolved and can easily be assigned to C_6_, C_3_, and C_9/9′_. These experiments taken together highlight the versatility and compatibility of OE‐DNP with the conditions required in high‐resolution liquid‐state NMR spectroscopy.

### Analysis of ^19^F OE‐DNP Mechanism

**Figure 4:**
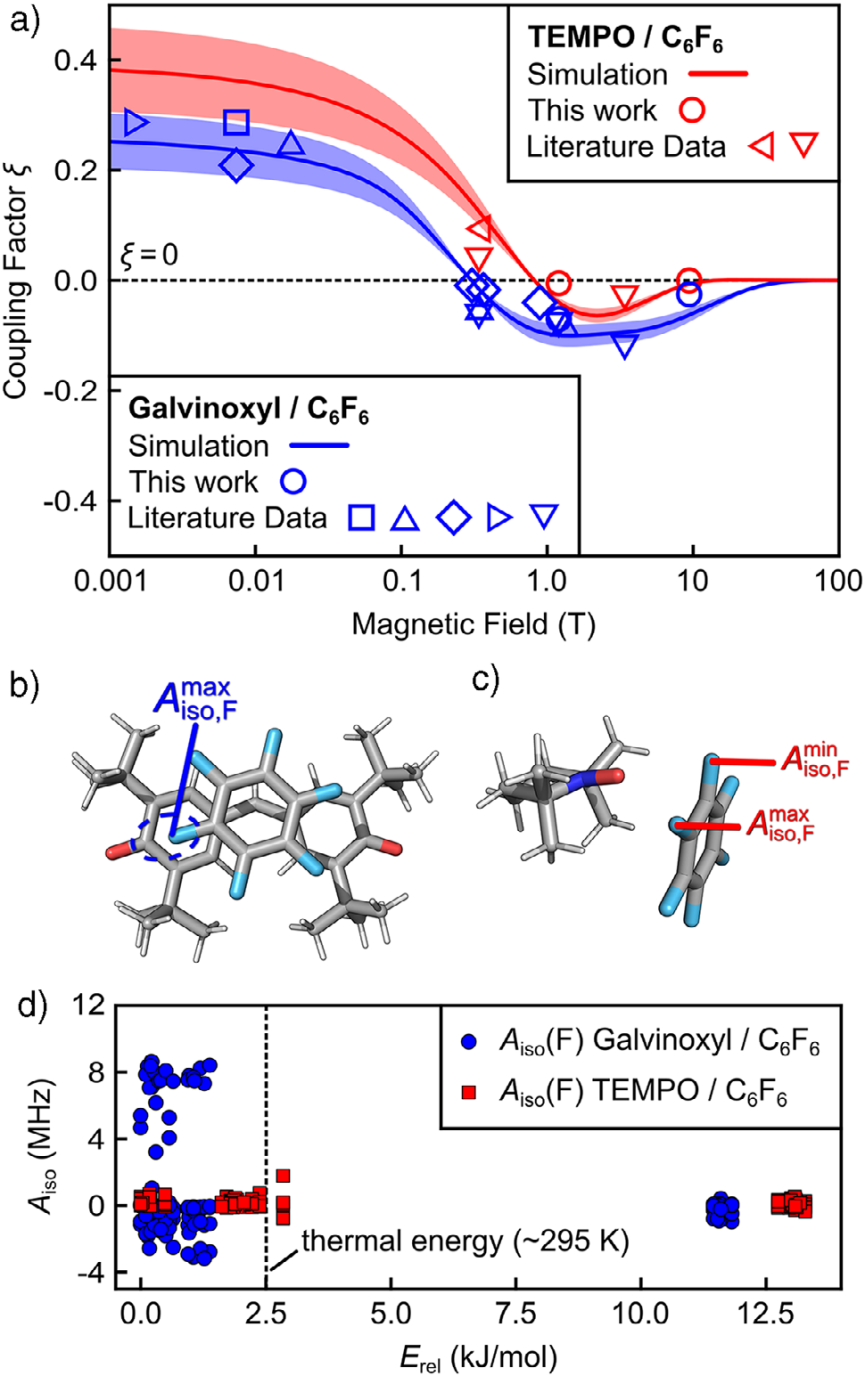
a) Magnetic field dependence of the coupling factor *ξ* of TEMPO/C_6_F_6_ (red) and galvinoxyl/C_6_F_6_ (blue). Lines are calculations using Equation S3. For TEMPO/C_6_F_6_ we used τ_D_ = 50 ps and τ_sc_ = 1.0 ps, while for galvinoxyl/C_6_F_6_ τ_C_ = 40 ps, τ_D_ = 50 ps, τ_sc,1_ = 4.1 ps, and τ_sc,2_ = 0.3 ps were used. A complete list of all simulation parameters is provided in Table S2. The colored areas represent a ± 20% uncertainty range around the simulation. Different symbols represent experimental data from: George and Chandrakumar^[^ ^31^ ^]^ ▾, Neudert et al.^[^ ^32^ ^]^ ◂, Peksoz et al.^[^ ^33^ ^]^ ▸, Webb et al.^[^ ^34^ ^]^ ⧫, Müller‐Warmuth et al.^[^ ^35^ ^]^ ▴, Poindexter et al.^[^ ^36^ ^]^ ▪, and this work ●. Data and errors are provided in the last column of Table S1. Errors on *ξ* are in the range of 3%–40% and are omitted in the plot for clarity. b), c) DFT optimized structures with maximum *A*_iso_ of galvinoxyl/C_6_F_6_ (b, *A*_iso_(F) ≈ 8.6 MHz, *E*_rel_ ≈ 0.22 kJ mol^−1^) and TEMPO/C_6_F_6_ (c, *A*_iso_(F) ≈ 1.8 MHz, *E*_rel_ ≈ 2.9 kJ mol^−1^). d)The hfc *A*_iso_ of the PA to ^19^F of C_6_F_6_ calculated for all optimized structures of the complexes galvinoxyl/C_6_F_6_ (blue) and TEMPO/C_6_F_6_ (red) plotted against the relative single‐point energy of the PA/C_6_F_6_ complex. Errors of *A*_iso_ are ∼15%, and error bars are omitted for clarity. Data shown in the figure were partially reported in the PhD thesis of one author.^[^ ^86^ ^]^^.^

These results open up the question of whether the observed enhancements can be rationalized within the framework of the available OE‐DNP theory and can be expected at even higher magnetic fields. To address this question, we have analyzed the magnetic field dependence of ^19^F coupling factors (Equation S3), determined using the Overhauser equation^[^ ^56^ ^]^

(1) $$\varepsilon =1-\frac{\left|\gamma_{e}\right|}{\gamma_{19F}}sf\xi$$

where *γ*_e_ and *γ*_19F_ are the gyromagnetic ratios of the electron spin and ^19^F, respectively, *s* is the saturation factor, *f* is the leakage factor, and *ξ* is the coupling factor. While *s* describes the degree of saturation of the PA through MW, *f* reflects to what extent nuclear relaxation is driven by the paramagnetic PA. The coupling factor ξ contains information on the polarization transfer mechanism via cross‐relaxation. By estimating *f* and *s* independently as described in Sections S4 and S5, we were able to estimate ξ from our ^19^F enhancements at 9.4 and 1.2 T as well as from literature data at magnetic fields ranging from 1.5 mT to 3.4 T.^[^ ^31^, ^32^, ^33^, ^34^, ^35^, ^36^ ^]^ All these data are summarized in Table S1. For a better understanding of the mechanism, we compared two systems: galvinoxyl/hexafluorobenzene (C_6_F_6_), for which we obtained at 9.4 T an enhancement of *ε* = 16 ± 3 (Figure S3a), and TEMPO/C_6_F_6_, where TEMPO is much less effective (*ε* = 1.0 ± 0.2, Figure S3b). Similarly, ^19^F enhancements obtained using nitroxides instead of galvinoxyl were considerably reduced for fluorobenzene **15** and fluoropentane **6** (Figure S4). In Figure 4a, we plotted the coupling factors of TEMPO/C_6_F_6_ (red) and galvinoxyl/C_6_F_6_ (blue) as a function of the magnetic field. For both systems, *ξ* is positive at low magnetic field (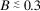 T), then turns negative with increasing magnetic field and remains of similar magnitude in the range ∼1–10 T. The behavior is in line with previously reported models that OE‐DNP enhancements at high fields are driven by subpicosecond molecular collisions, which modulate the scalar part of the hfc, resulting in a negative ξ (Equation S2).^[^ ^91^, ^92^ ^]^

We utilized the analytical model for the coupling factor (Section S5 and Equation S3) to simulate the field dependence in Figure 4a.^[^ ^92^, ^93^ ^]^ The relevant time scales entering scalar relaxation are included in the spectral density function Equation S9. The simulations for TEMPO/C_6_F_6_ were performed with a correlation time of τ_sc_ ≈ 1.0 ps. However, to simulate the plateau of ξ (1–10 T) observed in galvinoxyl/C_6_F_6_, two correlation times were required, one in the picosecond scale (τ_sc,1_ ≈ 4 ps) and one in the sub‐picosecond range (τ_sc,2_ ≈ 0.3 ps). Also, the amplitude of the spectral density ($F=\frac{\left\langle A^{2}\right\rangle }{\hbar^{2}\tau_{p}}$, Equation S9) for the fastest dynamic contribution is larger for galvinoxyl than for TEMPO, i.e., *F*_galv_ = 1.68 × 10^25^ (rad^2^ s^−2^) and *F*_TEMPO_ = 1.69 × 10^24^ (rad^2^ s^−2^), Table S2. This is consistent with larger intermolecular hfc in the transient complex of C_6_F_6_ with galvinoxyl with respect to TEMPO, as will be illustrated below. More details on the simulation parameters are given in Table S2. Importantly, the simulation predicts significant ξ(^19^F) for *B*_0_ ≫ 10 T opening the door for OE‐DNP at even higher fields. Consistent with previous reports,^[^ ^60^ ^]^ a rotational diffusion contribution can be neglected for the TEMPO/C_6_F_6_ system, while it is included for galvinoxyl (with τ_C_ = 40 ps). The value of τ_C_ is comparable to the rotational correlation time of galvinoxyl in toluene estimated by EPR measurements (Section S7) as well as to the reported value of galvinoxyl in toluene.^[^ ^94^ ^]^

To gain more insights into the molecular details,^[^ ^64^, ^65^, ^66^, ^95^ ^]^ we used DFT to calculate relative energies of transient complexes and related intermolecular hfcs *A*_iso_ for a total of 36 galvinoxyl/C_6_F_6_ and 31 TEMPO/C_6_F_6_ complexes. Details of the calculations are given in Section S6. The hfcs for all calculated structures are given in Figure 4d, where *A*_iso_ is plotted against the relative single‐point energy of each conformation. We find that, among the accessible conformations at room temperature, *A*_iso_ can attain larger values for galvinoxyl (up to ∼9 MHz) than for TEMPO (up to ∼2 MHz). This is in agreement with the simulation of the field dependence of ξ, where we employed *F*_galv_ ≫ *F*_TEMPO_. A view into the two complex structures with maximum *A*_iso_ and relative energies close to thermal energies (Figure 4b,c) shows differences between the complexes TEMPO/C_6_F_6_ and galvinoxyl/C_6_F_6_. This could be a result of the electrostatic repulsion between the electron‐rich NO group of TEMPO and the electronegative ^19^F atoms of C_6_F_6_ prevents electron spin density delocalization or spin polarization from the radical to the ^19^F nucleus. On the contrary, galvinoxyl and C_6_F_6_ stack with the aromatic rings parallel to each other (Figure 4b), which allows for a more efficient orbital overlap. More precise calculations at a higher level of theory are in progress and might enable more mechanistic insights into the electronic structure of these complexes as well as predict optimal OE‐DNP on specific chemical sites and other PAs.

## Conclusion

In this manuscript, we reported OE‐DNP hyperpolarized ^19^F NMR spectra on a variety of fluorinated molecules at a magnetic field, i.e, relevant for high‐resolution NMR spectroscopy. ^19^F NMR signal enhancements on small molecules are about one order of magnitude, which translates to two orders of magnitude gain in experiment time. These enhancements are not limited to small aromatic building blocks but were observed in a range of small molecules, including amino acids and drug compounds. ^19^F resonances of sample amounts as low as ∼2–5 nmol were readily detectable after 30–60 min of signal averaging.

In a second key step, we moved further and demonstrated how this can serve as an exquisite source of hyperpolarization of scalar‐coupled ^13^C nuclei at natural abundance and within the same molecule. We used ^19^F→^13^C OE‐DNP INEPT experiments, in which the hyperpolarization on ^19^F is transferred to ^13^C with an additional amplification factor given by γ19F/γ19Fγ13Cγ13C≈3.7. This resulted in up to 100‐fold increase in ^13^C signals *J*‐coupled to fluorine. Particularly, on a representative fluorinated drug molecule, ^19^F polarization could be transferred to nearby quaternary ^13^C nuclei, which are challenging to detect in standard NMR as well as by direct ^13^C OE‐DNP. ^13^C spectra without the ^19^F hyperpolarization step were not detectable even after hours of acquisition at comparable experimental conditions. We note that an analogous strategy using ^1^H instead of ^19^F is not effective, as direct ^1^H OE‐DNP enhancements at high fields are poor. Therefore, our results establish ^19^F as an unprecedented hyperpolarization source for the nuclear network. This, combined with the capability to perform 2D NMR experiments, renders OE‐DNP a general approach for small molecule structure elucidation. We anticipate that the combination of OE‐DNP with cryo‐probe technology could yield another up to four‐fold increase in sensitivity.^[^ ^96^ ^]^

In addition to the experimental endeavor, we provide a mechanistic interpretation of the OE‐DNP data over a range of 10 T for two representative systems, galvinoxyl/C_6_F_6_ and TEMPO/C_6_F_6_. This reveals a very weak field dependence above 1 T, opening the perspective of extending these experiments even at higher magnetic fields, for instance, 14 T. For these model systems, similar to what occurs for ^13^C, molecular dynamics in the sub‐picosecond time scale and large hfcs are essential for efficient OE‐DNP at high magnetic fields. The galvinoxyl radical fulfills these requirements, making it a suitable PA for ^19^F OE‐DNP. However, we foresee that other PAs might be identified in the future, which deliver larger spin density transfer to ^19^F, thus boosting the OE‐DNP performance.

The presented investigation, which combines ^19^F OE‐DNP screening, ^19^F→^13^C hyperpolarization transfer experiments, and mechanistic analysis, establishes ^19^F as one of the most promising nuclei for NMR OE‐DNP hyperpolarization in solution at high magnetic fields. OE‐DNP enhanced ^19^F NMR will provide new opportunities, for example, for studying the metabolic fate of pharmaceuticals, where ^19^F is often introduced to increase their metabolic stability, without negatively affecting their intended pharmacological action.

## Supporting Information

The authors have cited additional references within the Supporting Information.^[^ ^97^, ^98^, ^99^, ^100^, ^101^, ^102^, ^103^, ^104^, ^105^, ^106^, ^107^, ^108^, ^109^, ^110^, ^111^, ^112^, ^113^, ^114^, ^115^, ^116^, ^117^, ^118^, ^119^, ^120^ ^]^ S1: General Sample Preparation, S2: Experimental setup, methods and ^19^F and ^13^C OE‐DNP NMR spectra at 9.4 T; S3: Polarization transfer experiments at 9.4 T under OE‐DNP conditions; S4: Experimental setup, methods and ^19^F OE‐DNP NMR spectra at 1.2 T; S5: Coupling factors from ^19^F OE‐DNP enhancements and simulation parameters; S6: Quantum chemical calculations; S7: EPR characterization of galvinoxyl radical at 9.4 Tesla; S8: References.

## Conflict of Interests

The authors declare no conflict of interest.

## Supporting information



Supporting Information

## Data Availability

All original data will be made freely available at the Göttinger Data Repository GRO.data under the link: https://doi.org/10.25625/JCYI4Y.
