# Supplementary material for: Enhancing NMR Signals in Liquids by Fluorine‐19 Overhauser Dynamic Nuclear Polarization (DNP) and Hyperpolarization Transfer to Carbon‐13
Source: Angew Chem Int Ed Engl. 2025 Oct 23;64(50):e202517498. doi: 10.1002/anie.202517498 (PMC12684320; doi:10.1002/anie.202517498)
Supplement: Supplementary file 1 — Supporting Information [file ANIE-64-e202517498-s001.pdf]

Supporting Information

Enhancing NMR Signals in Liquids by Fluorine-19 Overhauser Dynamic Nuclear Polarization (DNP) and Hyperpolarization Transfer to Carbon-13

Maik Reinhard,[a,b] Alex van der Ham, [a] Luming Yang, [a] Leonard Bröker, [a,b] Tomas Orlando,[a, c] Igor Tkach,[a] Marcel Levien,[a,d],* and Marina Bennati.[a,b],*

Table of Contents

S1: General sample preparation 2
S2: Experimental setup, methods and ^{19}F and ^{13}C OE-DNP NMR spectra at 9.4 T 2
S3: Polarization transfer experiments at 9.4 T under OE-DNP conditions 6
S4: Experimental setup, methods and ^{19}F OE-DNP NMR spectra at 1.2 T 10
S5: Coupling factors from ^{19}F OE-DNP enhancements and simulation parameters 13
S6: Quantum chemical calculations 16
S7: EPR characterization of galvinoxyl radical at 9.4 Tesla 18
S8: References 20

S1: General sample preparation

1,4-Difluorobenzene, 4-fluoroanisole, 4-fluroacetophenone, 1-fluoronaphthalene, 3-chloro-4-fluoronitrobenzene, 1-fluoropentane, benzoylfluoride, methyl trifluoroacetate, flutamide, 2,3,4,6-tetra-O-acetyl-α-D-glucopyranosyl fluoride, L-Fmoc-3-fluorophenylalanine, hexafluorobenzene, fluorobenzene, 2,6-di-tert-butyl-α-(3,5-di-tert-butyl-4-oxo-2,5-cyclohexadien-1-ylidene)-*p*-tolyloxy (galvinoxyl, commercial radical purity was ~22%), 2,2,6,6-tetramethylpiperidin-1-oxyl (TEMPO) and flutrimazole were purchased from Sigma Aldrich. Benzoylfluoride was purchased from abcr GmbH, 2,3,4,6-tetra-O-acetyl-α-D-glucopyranosyl fluoride from BLD Pharmatech GmbH, (3-bromophenyl)sulfur pentafluoride and galvinoxyl (for experiments in Fig. 1d, Fig. 3, Fig. S7b, and Fig. S10-12, commercial radical purity was ~89%) from TCI Deutschland GmbH, while L-Fmoc-3-fluorophenylalanyl-L-3-fluorophenylalanine methyl ester was synthesized by the facility for synthetic chemistry of the Max Planck Institute for Multidisciplinary Sciences in Göttingen using protocols from the literature.[1, 2] Organic solvents tetrachloromethane (CCl_4), chloroform ($CHCl_3$) and dimethyl sulfoxide (DMSO) were purchased from Merck KGaA. All chemicals were used as received.

Commercial ~5.0 mm O.D. clear fused quartz NMR tubes (Wilmad-LabGlass) were used for 9.4 T DNP samples. Solutions of 60 – 75 µL containing ~0.1 – 500 mM of the target molecule and ~10 – 25 mM of polarizing agent were degassed by at least five freeze-pump-thaw cycles. A smaller ~4 mm O.D. quartz tube was inserted into the larger 5 mm NMR tube inside a glovebox (MBraun, nitrogen filled) confining the solution into a thin layer and the tube was closed with in-house made air-tight caps. For ^{19}F OE-DNP measurements at 1.2 T, the target served as the solvent using ~10 mM PA. The samples were degassed by 3 – 5 freeze-pump-thaw cycles in stock solutions (~100 – 200 µL) and ~4 – 7 µL were filled into a 1.6 mm O.D. and 1.1 mm I.D. Q-band quartz tube (Wilmad-LabGlass), which was sealed with a flame. The PA concentration and sample degassing were verified by CW EPR spectra (X-band, Bruker ElexSys E500T, ElexSys high sensitivity probe). The experimental error of the radical and target molecule concentrations is estimated to be 10 – 20%, the radical concentration was stable for the duration of the measurements.

S2: Experimental setup, methods and ^{19}F and ^{13}C OE-DNP NMR spectra at 9.4 T

The 9.4 T DNP setup consists of a commercial NMR magnet (Bruker wide-bore Ultrashield, Bruker Avance Neo NMR console) equipped with a frequency-tunable gyrotron (4.8 T cryogen-free magnet, ~263.30 ± 0.25 GHz with output power up to ~50 W), which is connected via a corrugated waveguide to the probe. A modified commercial two-channel high-resolution wide-bore liquid NMR probe by Bruker is used for simultaneous RF and MW irradiation. The setup was described in detail in the literature.[3] The experiments were performed under slow spinning of the sample (20 Hz) and the sample temperature was controlled by a cold nitrogen gas flow under MW irradiation resulting in an effective sample temperature of ~300 K. Nevertheless, we observed an NMR line broadening under MW irradiation, due to the temperature-sensitive chemical shift of ^{19}F. The linewidth in Figure 1c increased from ~50 Hz without MW irradiation to ~80 Hz under MW irradiation, while in other cases the bottom of the signal was inhomogeneously broadened (Figure S1 – S4). MW irradiation was applied on resonance of the

EPR transition of the respective radical by adjusting gyrotron parameters to tune its frequency. The MW power was ~40 – 50 W during the DNP experiments depending on the PA and solvent. RF pulse lengths of t_p(RF) = 16.5 – 17 µs with a power of 21 W was used for a 90° pulse. Interscan delays were usually set to 5 – 10 s. Depending on the signal intensity of the respective sample 4 – 64 scans were acquired with MW irradiation while 4 – 2048 scans were used for experiments without MW irradiation. Polarization transfer experiments (9.4 T, Fig. 2, Fig. 3, Fig. S7, Fig. S8, Fig. S11, Fig S12) used INEPT and HETCOR pulse sequences (see Fig. S5).

The 1D ^{19}F NMR spectra at 9.4 T were measured using a pulse acquire pulse sequence consisting of a 90° RF pulse that was calibrated before (t_p = 16.5 – 17 µs) followed by FID detection. This sequence was with recycle delay (RD) for ~5T_{1n} allowing for thermal polarization recovery. For the OE-DNP experiments, CW MW irradiation was applied throughout the sequence. The spectra were processed using TopSpin (ver. 4.4.0) and MestReNova (ver. 11.0.4). Phase and baseline correction were performed after Fourier transformation. The enhancements were calculated by the ratio of the integrated NMR signals with (I_{DNP}) and without ($I_{thermal}$) MW irradiation corrected by the number of scans (ns_{DNP} and $ns_{thermal}$) as $\varepsilon = (I_{DNP} \cdot ns_{thermal})/(I_{thermal} \cdot ns_{DNP})$ and the errors of the enhancements are estimated to be 15%. Longitudinal nuclear relaxation times were determined with inversion-recovery experiments (180° RF pulse, delay τ, 90° RF pulse, FID detection). The delay τ is incremented, the NMR signal is plotted as function of the delay and the nuclear relaxation times T_{1n} and T_{1n}^0 with and without the presence of the PA, respectively, were determined from mono exponential fits (Fig. S15b-d). Data shown in Fig. S1-S4 were partially reported in the PhD thesis of one author.[4]

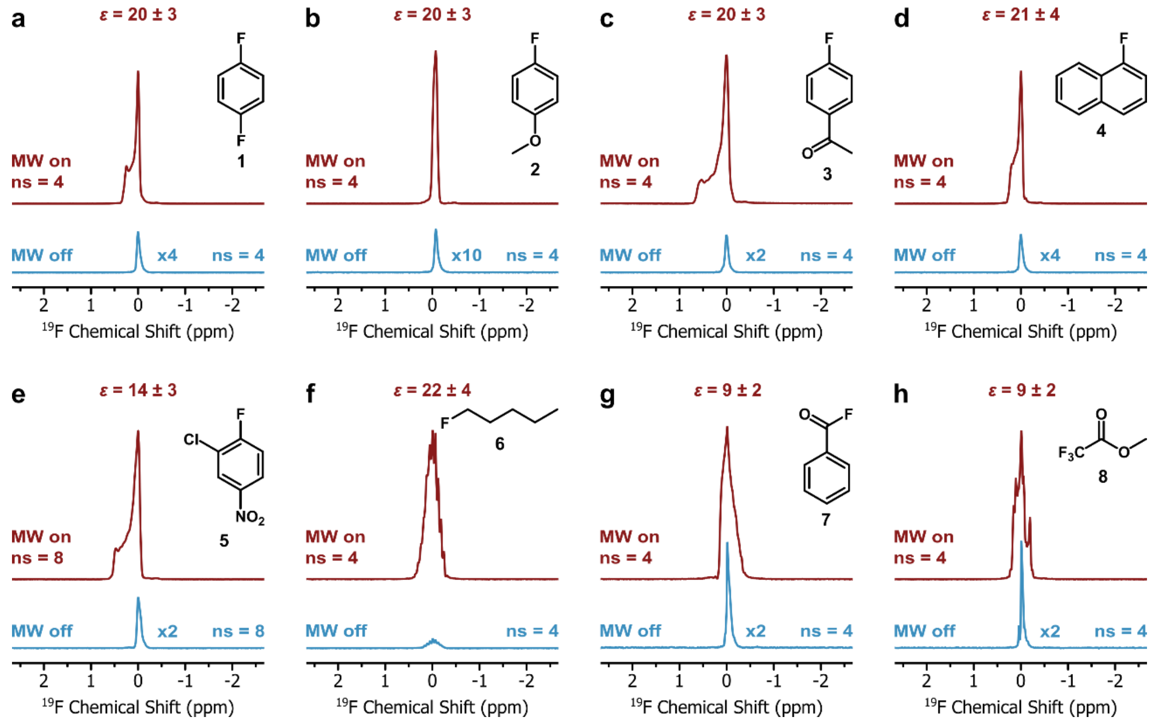

Figure S1: 1D ^{19}F NMR signal enhancements of small fluorinated molecules at 9.4 T shown in Fig. 1a in the main text. ^{19}F OE-DNP (red) and Boltzmann (blue) spectra of **a)** 1,4-difluorobenzene, **b)** 4-fluoroanisole, **c)** 4-fluoroacetophenone, **d)** 1-fluoronaphthalene, **e)** 3-chloro-4-fluoronitrobenzene, **f)** 1-fluoropentane, **g)** benzoylfluoride, and **h)** methyl trifluoroacetate. The target molecule concentration of all samples was ~500 mM and they were doped with ~10 mM of galvinoxyl as polarizing agent dissolved in CCl_4. The chemical shifts were set to 0 ppm due to lack of an internal standard. The error of the

enhancements is assumed to be 15%. ^{19}F NMR/DNP experimental parameters: $T \approx 300$ K, $V_{sample} \approx 20$ µL, $P_{MW} \approx 50$ W (CW), t_p(90° RF) = 16.5 – 17 µs, P_{RF} = 21 W, RD = 10 s, LB = 3 Hz, number of scans are displayed in the figure.

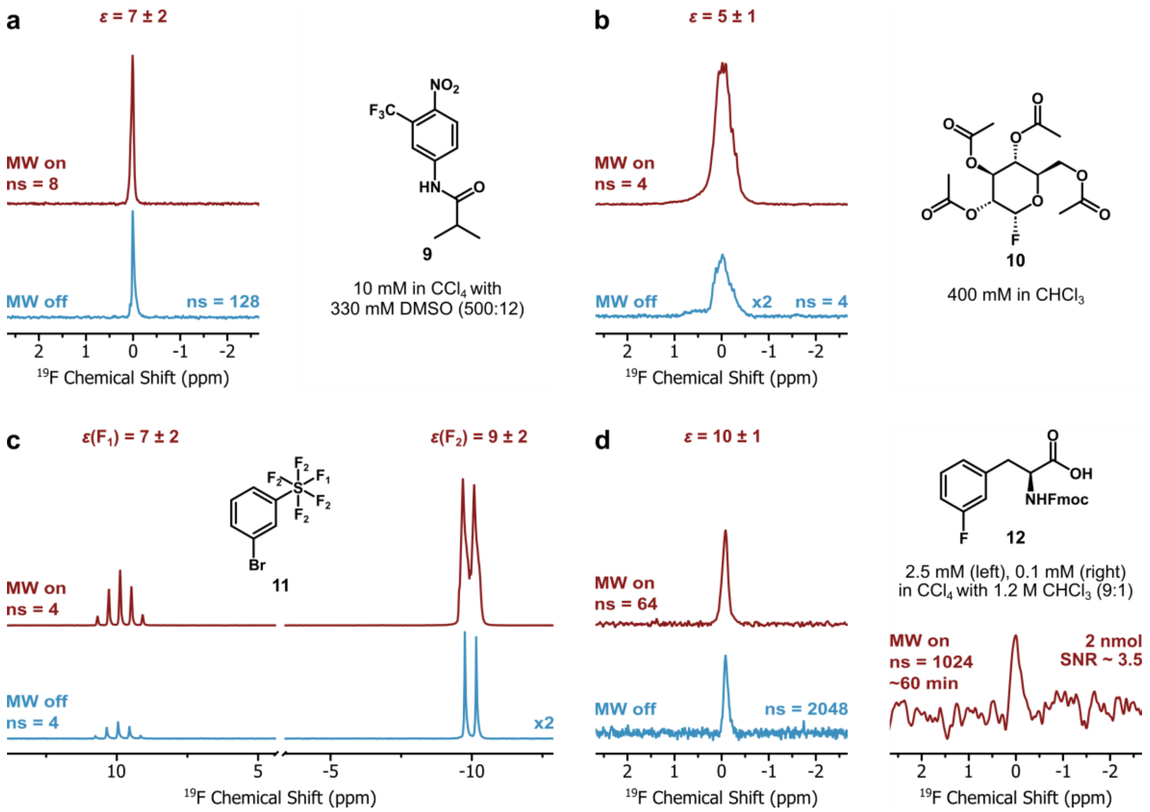

Figure S2: 1D ^{19}F NMR signal enhancements of selected fluorinated molecules at 9.4 T shown in Fig. 1b in the main text. ^{19}F OE-DNP (red) and Boltzmann (blue) spectra of **a)** flutamide ($c \approx 10$ mM in CCl_4/DMSO, 500/12, v/v),[3] **b)** 2,3,4,6-tetra-O-acetyl-α-D-glucopyranosyl fluoride ($c \approx 400$ mM in $CHCl_3$), **c)** (3-bromophenyl)sulfur pentafluoride ($c \approx 500$ mM in CCl_4), and **d)** ʟ-Fmoc-3-fluorophenylalanine ($c \approx 2.5$ mM (left) and $c \approx 0.1$ mM (right) in CCl_4/$CHCl_3$, 9/1, v/v). The samples were doped with ~10 mM of galvinoxyl as polarizing agent. The chemical shifts were set arbitrary due to lack of an internal standard. The error of the enhancements is assumed to be 15%. ^{19}F NMR/DNP experimental parameters: $T \approx 300$ K, $V_{sample} \approx 20$ µL, $P_{MW} \approx 50$ W (CW), t_p(90° RF) = 16.5 – 17 µs, P_{RF} = 21 W, RD = 2.5 – 10 s, LB = 3 Hz (30 Hz in **d** right), number of scans are displayed in the figure.

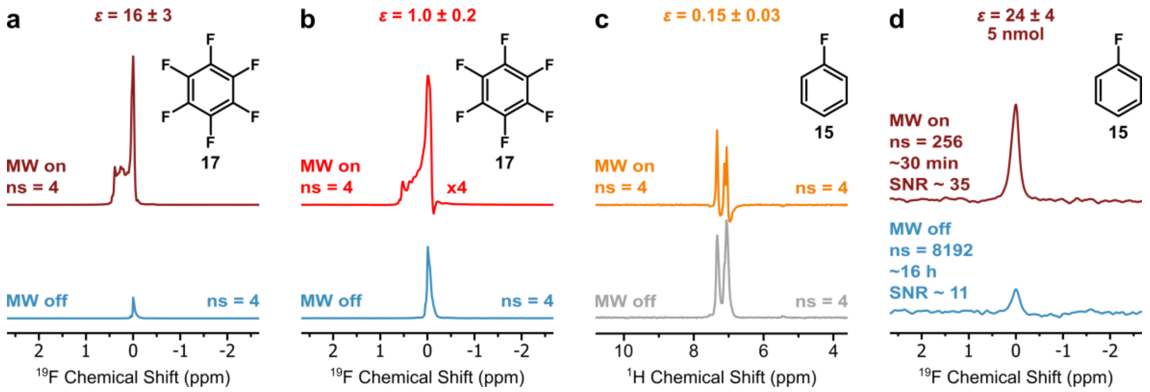

Figure S3: **a**, **b)** 1D ^{19}F NMR signal enhancements of the investigated fluorinated model system using different PAs at 9.4 T shown in Fig. 4 in the main text. OE-DNP (red) using galvinoxyl (**a**) and TEMPO (**b**) and Boltzmann (grey) spectra of hexafluorobenzene ($c \approx 500$ mM in CCl_4). Samples were doped with ~10 mM of galvinoxyl (**a**) ~25 mM of TEMPO (**b**) as polarizing agents. The chemical shifts were set to 0 ppm due to lack of an internal standard. **c)** 1D ^{1}H NMR signal enhancement (orange) of fluorobenzene ($c \approx 500$ mM in CCl_4) using ~10 mM of galvinoxyl as PA and Boltzmann spectrum (grey). **d)** 1D ^{19}F NMR signal enhancement of ~5 nmol fluorobenzene ($c \approx 0.25$ mM in CCl_4) doped with ~15 mM of galvinoxyl as PA at 9.4 T (RD = 6 s, LB = 35 Hz). The error of the enhancements is assumed to be 15%. The spectra of **c)** were already reported.[3] ^{19}F

NMR/DNP experimental parameters: $T \approx 300$ K, $V_{sample} \approx 20$ µL, $P_{MW} \approx 50$ W (CW), t_p(90° RF) = 16.5 – 17 µs, $P_{RF} = 21$ W, RD = 10 s, LB = 3 Hz, number of scans are displayed in the figure.

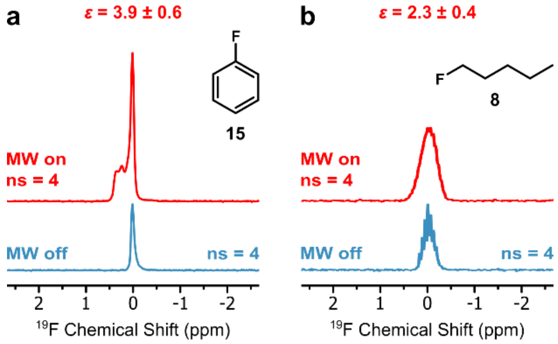

Figure S4: 1D ^{19}F NMR signal enhancements of small fluorinated molecules using TEMPO (**a**) and TEMPONE-^{15}N-d_{16} (**b**) as polarizing agents at 9.4 T. ^{19}F OE-DNP (red) and Boltzmann (blue) spectra of **a)** fluorobenzene, and **b)** 1-fluoropentane. The target molecule concentration of all samples was ~500 mM and they were dissolved in CCl_4 and doped with ~25 mM of PA. The chemical shifts were set to 0 ppm due to lack of an internal standard. The error of the enhancements is assumed to be 15%. ^{19}F NMR/DNP experimental parameters: $T \approx 300$ K, $V_{sample} \approx 20$ µL, $P_{MW} \approx 40$ W (CW), t_p(90° RF) = 16.5 – 17 µs, $P_{RF} = 21$ W, RD = 10 s, LB = 3 Hz, number of scans are displayed in the figure.

S3: Polarization transfer experiments at 9.4 T under OE-DNP conditions

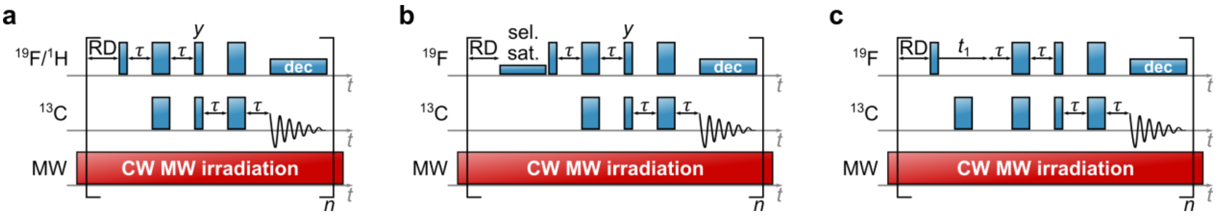

Figure S5: Pulse sequence of refocused INEPT (**a**), refocused INEPT with selective pre-saturation (sel. sat., **(b)**, and 2D HETCOR for polarization transfer experiments with decoupling (dec) of the source spin ($^{19}F/^{1}H$) and under CW MW irradiation **(c)**. Indicated are 90° and 180° pulses with shorter and longer rectangles, respectively. A recycle delay (RD) of ~$5T_{1n}$ is set before the sequence is repeated. The selective saturation pulse is in the order of seconds (**b**). The optimal value for the delay is $\tau = 1/4\,^{n}J_{C,F/H}$. The second dimension in the HETCOR originates from incrementing the time t_1 (**c**). All thermal INEPT and $^{1}H\rightarrow^{13}C$ INEPT experiments were measured without MW irradiation.

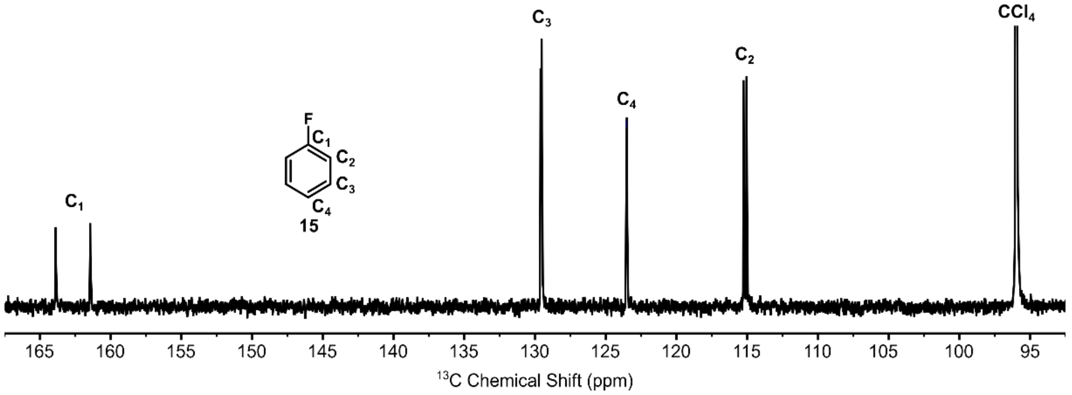

Figure S6: 1D ^{13}C pulse-acquire NMR spectrum of fluorobenzene with ^{1}H decoupling for $^{n}J_{C,F}$ coupling constants estimation. Approximately 500 mM of Fluorobenzene (natural ^{13}C abundance) was dissolved in ~400 µL CCl_4. ^{13}C NMR experimental parameters: T = 300 K, $V_{sample} \approx$ 400 µL, t_p(90° RF,C) = 10.5 µs, $P_{RF,C}$ = 41 W, $P_{RF,H,dec}$ = 0.6 W, RD = 30 s, LB = 1 Hz, ns = 1024. $^{13}C\{^{1}H\}$ NMR (100.4 MHz, CCl_4, ppm): δ = 162.66 (d, $^{1}J_{C,F}$ = 246.6 Hz, 1C; C1), 129.57 (d, $^{3}J_{C,F}$ = 7.7 Hz, 2C; C3, C3'), 123.53 (d, $^{4}J_{C,F}$ = 3.3 Hz, 1C; C4), 115.15 (d, $^{2}J_{CF}$ = 20.8 Hz, 2C; C2, C2'). Data shown in figure was initially reported in the PhD thesis of one author.[4]

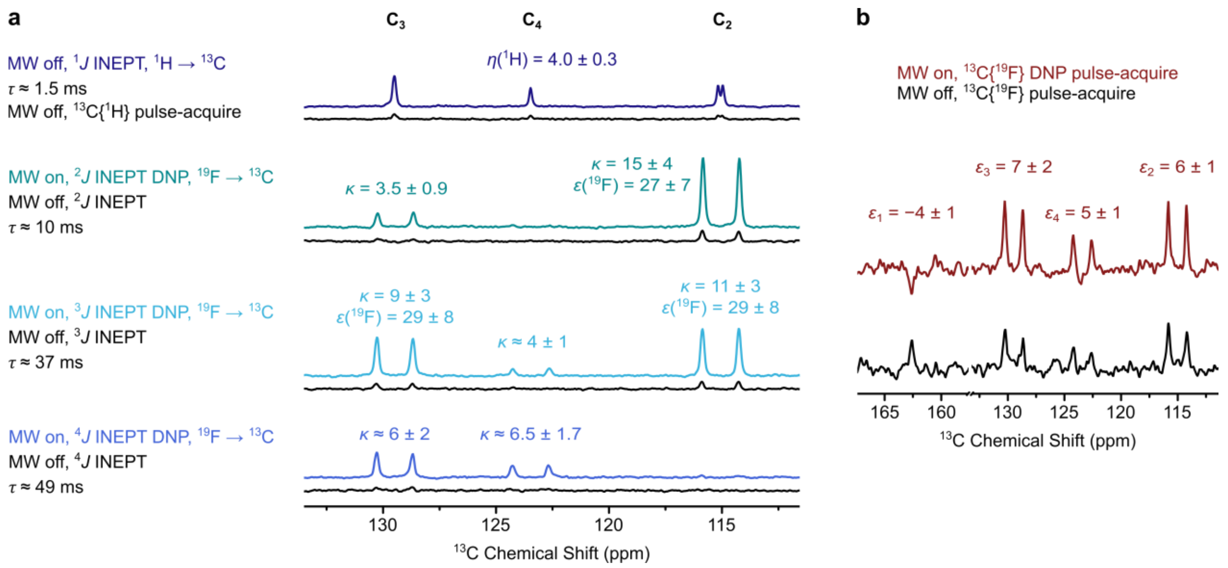

Figure S7: **a)** Comparison of thermal $^1H \rightarrow ^{13}C$ INEPT experiment (top) with $^{19}F \rightarrow ^{13}C$ DNP INEPT spectra and their thermal $^{19}F \rightarrow ^{13}C$ INEPT spectra (black) of model system fluorobenzene-C_2-C_4 (~500 mM, natural abundance) in CCl_4 doped with ~10 mM galvinoxyl using $\tau_{n,F/H} = 1/4^nJ_{C,F/H}$. Only the $^{19}F \rightarrow ^{13}C$ INEPTs were measured under MW irradiation and are from Figure 2d in the main text. All spectra were recorded with broadband decoupling of the source spin. The net enhancement κ compares the $^{19}F \rightarrow ^{13}C$ INEPT DNP spectra with the $^1H \rightarrow ^{13}C$ INEPT spectrum and is defined as $\kappa = \varepsilon(^nJ_{C,F}$ INEPT DNP$) / \eta(^1J_{C,H}$ INEPT) and the enhancement ε as $\varepsilon(^{19}F) = \varepsilon(^nJ_{C,F}$ INEPT DNP$)/ \eta(^nJ_{C,F}$ INEPT). The error of the enhancements is assumed to be 25%. **b)** Pulse-acquire $^{13}C\{^{19}F\}$ OE-DNP spectra of fluorobenzene (~500 mM, natural abundance) in CCl_4 doped with ~25 mM galvinoxyl with (red) and without (black) MW irradiation. NMR/DNP experimental parameters: $P_{MW} \approx 50$ W (CW), t_p(90° RF,C) = 10.5 µs, t_p(90° RF,F) = 17 µs, t_p(90° RF,H) = 14.7 µs, $\tau_{2\text{-}4,F} \approx$ 10, 37, 49 ms, $\tau_{1,H} \approx$ 1.5 ms, $P_{RF,C}$ = 41 W, $P_{RF,F}$ = 21 W, $P_{RF,F,dec}$ = 1.2 W, $P_{RF,H}$ = 21 W, $P_{RF,H,dec}$ = 1.2 W, RD(a) = 6 s, RD(b) = 30 s, LB(a) = 10 Hz, LB(b) = 20 Hz, ns($^1H \rightarrow ^{13}C$ INEPT) = 1280, ns($^{13}C\{^1H\}$) = 512, ns($^{19}F \rightarrow ^{13}C$ DNP INEPT) = 96, ns($^{19}F \rightarrow ^{13}C$ INEPT) = 1280, ns($^{13}C\{^{19}F\}$ DNP) = 64, ns($^{13}C\{^{19}F\}$ pulse-acquire) = 1280, exp. time: ~2.5 h for $^1H \rightarrow ^{13}C$ INEPT, ~4.5 h for $^{13}C\{^1H\}$ pulse-acquire, ~11 min for $^{19}F \rightarrow ^{13}C$ DNP INEPT, and ~2.5 h for $^{19}F \rightarrow ^{13}C$ INEPT spectra, ~0.5 h for ^{13}C DNP and ~11 h for $^{13}C\{^{19}F\}$ pulse-acquire. Data shown in figure were partially reported in the PhD thesis of one author.[4]

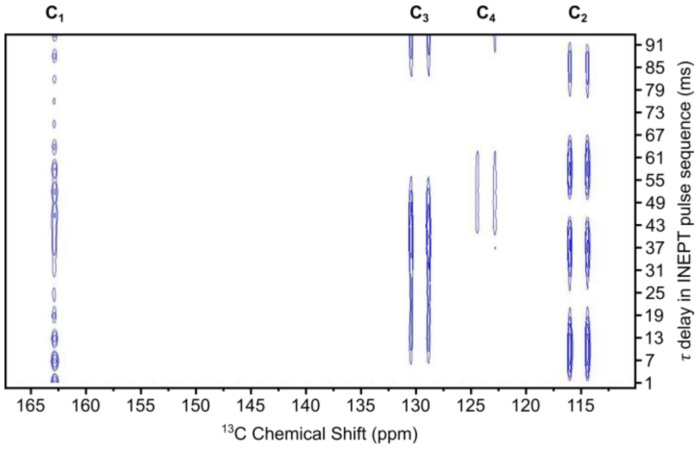

Figure S8: Contour plot of pseudo-2D DNP INEPT from Fig. 2c in the main text of fluorobenzene (500 mM) doped with ~10 mM galvinoxyl in CCl_4, where the delay τ in the INEPT sequence is incremented. The plot shows the oscillation of the intensity of the C_1-C_4 resonances as function of the delay. The initial delay is 1 ms and each increment is 3 ms. NMR/DNP experimental parameters: $P_{MW} \approx 50$ W (CW), t_p (90° RF,C) = 10.5 µs, t_p (90° RF,F) = 17 µs, $P_{RF,C}$ = 41 W, $P_{RF,F}$ = 21 W, $P_{RF,F,dec}$ = 1.2 W, RD = 6 s, LB = 10 Hz, ns = 96, exp. time: ~6 h.

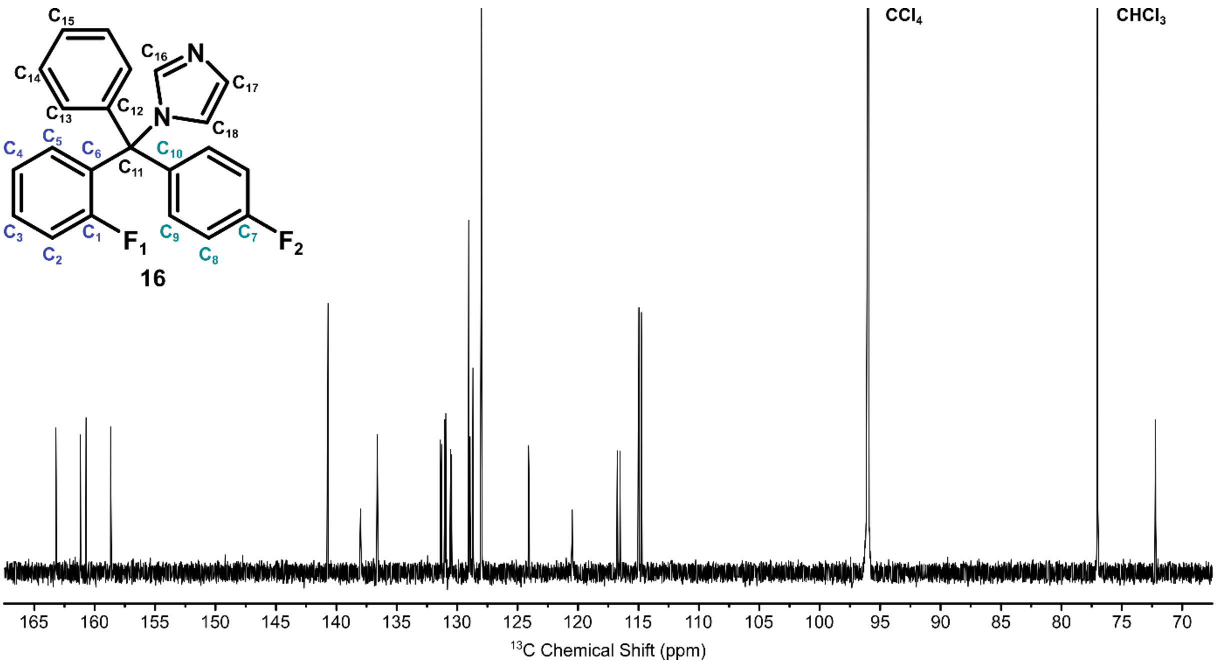

Figure S9: 1D ^{13}C NMR spectrum of flutrimazole with ^{1}H decoupling for $^{n}J_{C,F}$ coupling constants estimation. Approximately 125 mM of flutrimazole was dissolved in ~400 µL $CCl_4/CHCl_3$, 9/1, v/v. ^{13}C NMR experimental parameters: T = 300 K, V_{sample} ≈ 400 µL, t_p(90° RF,C) = 10.5 µs, $P_{RF,C}$ = 41 W, $P_{RF,H,dec}$ = 0.6 W, RD = 60 s, LB = 0 Hz, ns = 1024. $^{13}C\{^{1}H\}$ NMR (100.4 MHz, $CCl_4/CHCl_3$, 9/1, v/v, ppm): δ = 161.96 (d, $^{1}J_{C,F}$ = 250 Hz, 1C; C7), 159.93 (d, $^{1}J_{C,F}$ = 252 Hz, 1C; C1), 140.68 (s, 1C; C12), 138.00 (s, 1C; C16), 136.62 (d, $^{4}J_{C,F}$ = 3.6 Hz, 1C; C10), 131.33 (d, $^{2}J_{C,F}$ = 10.6 Hz, 1C; C6), 130.98 (d, $^{3}J_{C,F}$ = 7.8 Hz, 2C; C9, C9'), 130.50 (d, $^{3}J_{C,F}$ = 8.5 Hz, 1C; C3/C5), 129.04 (s, 2C; C14, C14'), 128.94 (d, $^{4}J_{C,F}$ = 2.6 Hz, 1C; C4), 128.69 (s, 1C; C17), 128.02 (s, 1C; C15) 127.99 (s, 2C; C13, C13'), 124.08 (d, $^{3}J_{C,F}$ = 3.4 Hz, 1C; C3/C5), 120.48 (s, 1C; C18), 116.63 (d, $^{2}J_{C,F}$ = 22.6 Hz, 1C; C2), 114.85 (d, $^{2}J_{C,F}$ = 21.4 Hz, 2C; C8, C8'), 72.22 (d, $^{3}J_{C,F}$ = 1.4 Hz, 1C; C11).

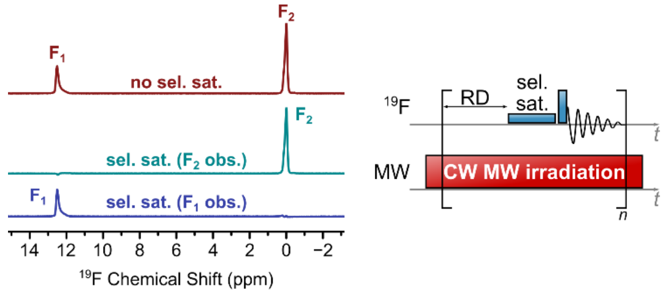

Figure S10: ^{19}F NMR spectra of flutrimazole under DNP conditions with ^{19}F pulse-acquire (red), selective ^{19}F pre-saturation of F_1 (F_2 observed, green) and selective ^{19}F pre-saturation of F_2 (F_1 observed, blue) to indicate the pre-saturation efficiency for the INEPT experiments (Fig. 3c and Fig. S11). The pulse sequence for these ^{19}F experiments is shown. ^{19}F NMR/DNP experimental parameters: Pulse-acquire and selective pre-saturation pulse-acquire sequence, P_{MW} ≈ 50 W (CW), t_p(90° RF,F) = 17 µs, t_p(RF,F,sat) = 0.5 s, $P_{RF,F}$ = 21 W, $P_{RF,F,sat}$ = 0.01 W, RD = 2.5 s, LB = 3 Hz, ns = 16, exp. time: ~1 min.

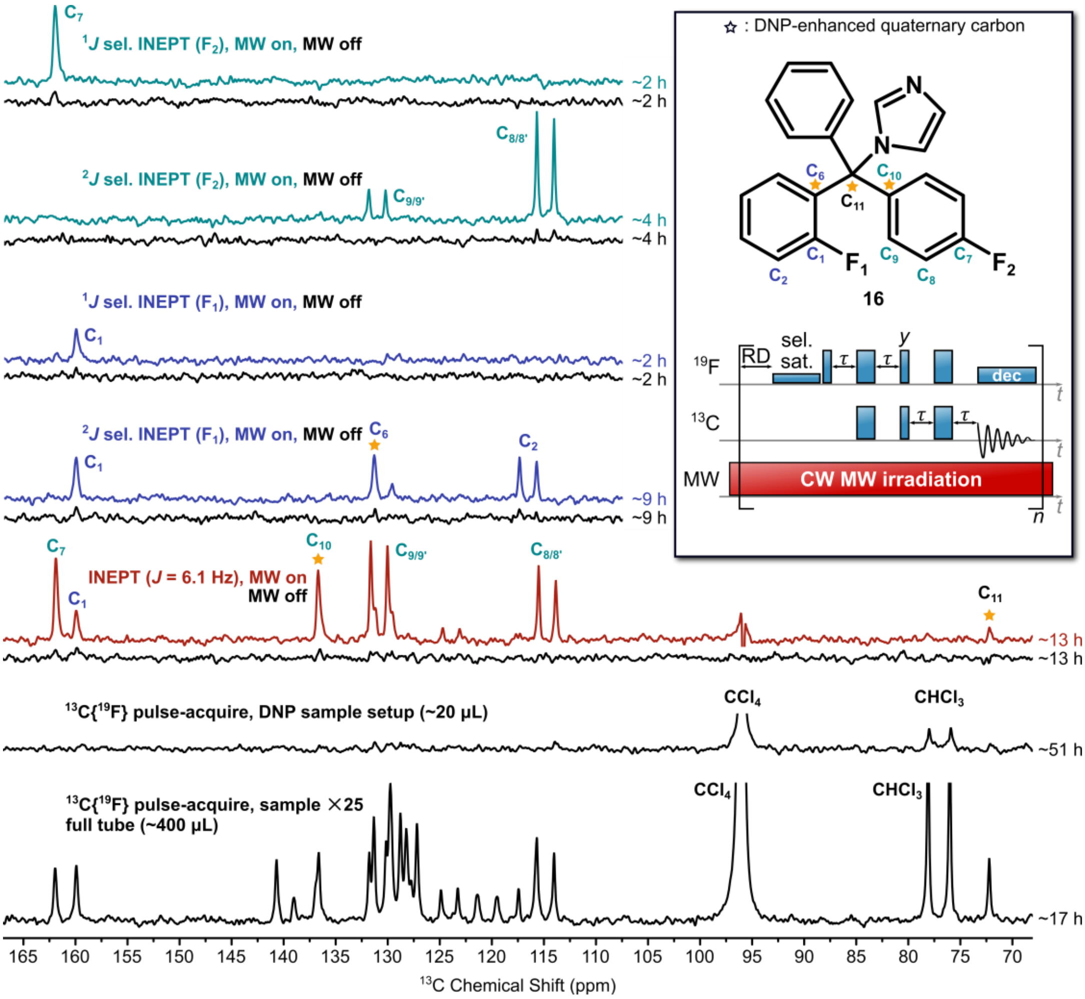

Figure S11: Extended Figure 3c from main text. $^{19}F\rightarrow^{13}C$ selective ^{19}F pre-saturated INEPT under MW irradiation of flutrimazole optimized for F_1 (blue) and F_2 (green) and their respective spectra without MW irradiation (black). The $^2J_{C,F}$ spectra are from Figure 3c in the main text. The inset shows the molecular structure of flutrimazole with fluorine and carbon labeling and the INEPT pulse sequence with selective pre-saturation (Fig. S5b). The red spectrum shows an INEPT (no pre-saturation) under DNP conditions and the respective spectrum without MW irradiation (black), where the delay tau τ was set to ~41 ms to detect the scalar coupled quaternary carbons C_{10} and C_{11}. The bottom two black spectra show ^{13}C pulse-acquire spectra without MW irradiation, where the top one was recorded with the DNP sample setup (~20 µL). The DNP sample contains ~100 mM of flutrimazole doped with ~15 mM galvinoxyl and dissolved in $CCl_4/CHCl_3$, 9/1, v/v. The bottom black spectrum shows a full tube (~400 µL) ^{13}C NMR spectrum of ~125 mM flutrimazole (~25-fold sample increase compared to DNP sample setup in $CCl_4/CHCl_3$, 9/1, v/v) as reference spectrum. All spectra were recorded with broadband ^{19}F decoupling. NMR/DNP experimental parameters: Selective pre-saturation INEPT, INEPT and pulse-acquire sequence, $P_{MW} \approx 50$ W (CW), t_p(90° RF,C) = 10.5 µs, t_p(90° RF,F) = 17 µs, t_p(RF,F,sat) = 0.5 s, $\tau_{1,F1} \approx 1$ ms, $\tau_{1,F2} \approx 1$ ms, $\tau_{2,F1} \approx 11$ ms, $\tau_{2,F2} \approx 11.7$ ms, $\tau_{red} \approx 41$ ms, $P_{RF,C}$ = 41 W, $P_{RF,F}$ = 21 W, $P_{RF,F,sat,DNP}$ = 0.01 W, $P_{RF,F,sat,Boltz}$ = 0.03 W, $P_{RF,F,dec}$ = 1.2 W, RD(INEPTs) = 2.5 s, RD(pulse-acquire) = 30 s, LB(^{13}C) = 20 Hz, ns(INEPT ($^1J_{C,F}$, F_1, F_2), MW on, MW off) = 2048, ns(INEPT ($^2J_{C,F}$, F_1), MW on, MW off) = 8192, ns(INEPT ($^2J_{C,F}$, F_2), MW on, MW off) = 4096, ns(INEPT, MW on, MW off) = 13312, ns(^{13}C pulse-acquire, ~20 µL) = 6000, ns(pulse-acquire, ~400 µL) = 1024, exp. time: ~2 h for ^1J INEPT, ~9 h for $^2J(F_1)$ INEPT, ~4 h $^2J(F_2)$INEPT, ~13 h for INEPT, ~51 h for ^{13}C pulse-acquire, ~20 µL and ~17 h for ^{13}C pulse-acquire, ~400 µL.

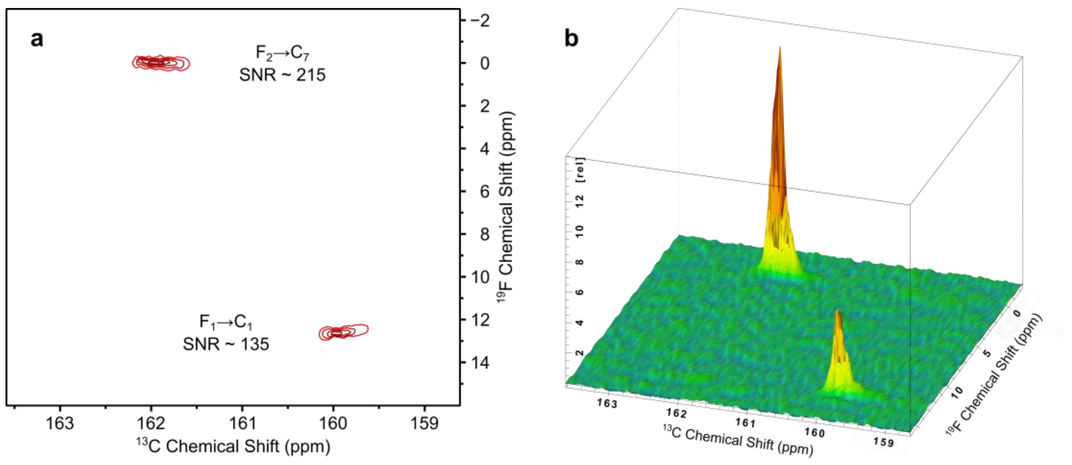

Figure S12: **a)** Contour plot and **b)** oblique plot of 2D $^{19}F \rightarrow ^{13}C$ HETCOR spectrum of flutrimazole with ^{19}F decoupling under DNP conditions. The sample composition is described in the caption of Fig. S11. The spectrum shows two cross-peaks correlating F_1 to C_1 and F_2 to C_7. NMR/DNP experimental parameters: HETCOR pulse sequence, $P_{MW} \approx 50$ W (CW), t_p(90° RF,C) = 10.5 µs, t_p(90° RF,F) = 17 µs, $\tau \approx 1$ ms, $P_{RF,C} = 41$ W, $P_{RF,F} = 21$ W, $P_{RF,F,dec} = 1.2$ W, RD = 1 s, ns = 1024, size of FID 512×32, exp. time: ~14 h. Processing parameters: Linear forward prediction, TDeff = 32, NCOEF = 2, LPBIN = 512 in F1.

S4: Experimental setup, methods and ^{19}F OE-DNP NMR spectra at 1.2 T

OE-DNP and EPR measurements at 1.2 T were performed on a hybrid setup consisting of a Bruker ElexSys E580 EPR spectrometer (34 GHz) combined with an AVANCE III NMR console. The sample was placed inside a Bruker cylindrical ER-5106QT/W CW resonator. A home-built copper coil for NMR detection and matching device for accurate matching and tuning was introduced, as reported previously.[5] The magnetic field was provided by a water-cooled electromagnet. A continuous wave (CW) travelling-wave-tube (TWT) amplifier (Applied Systems Engineering, Inc.) was used providing a MW power up to ~40 W. Saturation factors were obtained at 1.2 Tesla from pulsed ELDOR experiments (Fig. S14).[6] Pulse-acquire and inversion-recovery experiments were performed to determine the ^{19}F NMR signal enhancements (with and without MW) and nuclear longitudinal relaxation times, respectively.

To investigate the field dependence of the ^{19}F OE-DNP, we performed ^{19}F DNP at 1.2 T (setup described in the literature)[5] on hexafluorobenzene doped with galvinoxyl or TEMPO. The coupling factors were calculated with equation 1 from the main text (Overhauser equation). For this, the ^{19}F NMR signal enhancement ε, the saturation factor s and the leakage factor f were independently determined at 1.2 T.[5] The results of the measurements to determine these parameters are displayed in Figures S13 – S15a.

The ^{19}F NMR signal enhancements were measured using a pulse-acquire sequence as described in Sec S2 (90° RF pulse, FID detection, RD of ~5T_{1n}). A quasi CW MW pulse was applied before the RF pulse for the DNP experiments. The ^{19}F NMR signal enhancement was calculated as described in Sec S2. Nuclear relaxation times at 1.2 T were obtained from inversion-recovery experiments as described in Sec S2 (Fig. S13b, d and Fig. S15a). The leakage factor was calculated as $f = 1 - T_{1n}/T_{1n}^0$. The saturation factors were determined from electron-electron double resonance experiments (ELDOR). In the ELDOR experiment, a long MW saturation pulse (5 µs) to pump the electron spins. The frequency of this pulse is varied stepwise through the whole EPR spectrum of the radical. After the first pulse (with 8 ns delay), the EPR signal is measured with a 90° MW pulse (FID detection). The detection was fixed on the low field resonance line of galvinoxyl or TEMPO. When the saturation pulse is on resonance with either

one of the EPR transitions, the EPR signal intensity drops. The saturation factors s_i can be determined from these signal drops in the ELDOR spectra (Fig. S14b, d) and the effective saturation factor s_{eff} is calculated as the average of all s_i. All values are displayed in Table S1. The MW power used in the ELDOR experiments was the same as in the OE-DNP experiments. Data shown in Fig. S13-S15 were partially reported in the PhD thesis of one author.[4]

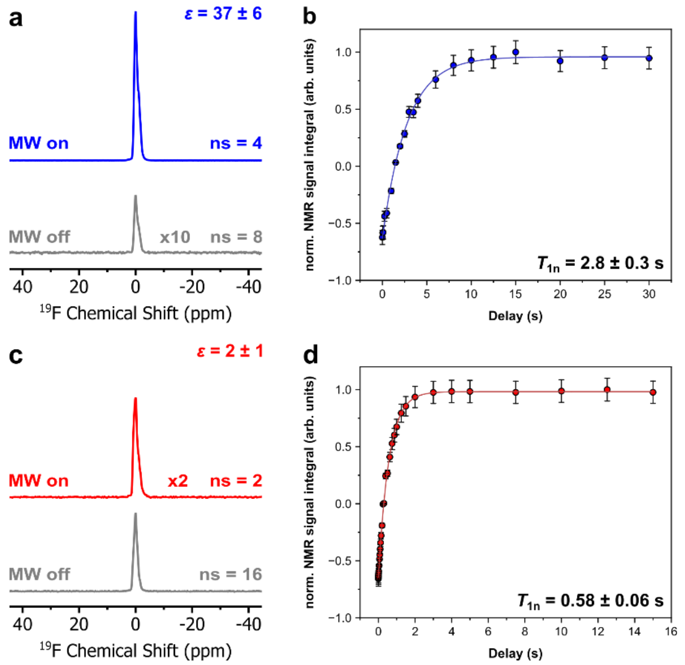

Figure S13: **a, c)** 1D ^{19}F OE-DNP enhanced and Boltzmann (grey) NMR spectra of hexafluorobenzene with galvinoxyl (blue) or TEMPO (red) as PA measured at 1.2 T. The error of the enhancement is assumed to be 15%. **b, d)** Plotted NMR signal integrals from inversion-recovery experiments and mono exponential fits to determine the ^{19}F nuclear relaxation times T_{1n} of hexafluorobenzene in presence of the radical at 1.2 T. The error of the NMR signal integral and the nuclear relaxation times are estimated to be 15%. Both samples employed ~10 mM of PA dissolved in neat hexafluorobenzene (~8.7 M). ^{19}F NMR/DNP experimental parameters: $T \approx 300$ K, $V_{sample} \approx 5$ µL, $P_{MW} \approx 40$ W (quasi CW, 7.5 s (galvinoxyl) and 0.5 s (TEMPO)), t_p(90° RF,F) = 3 – 3.5 µs, P_{RF} = 60 W, RD = 5 s, LB = 3 Hz and ns = 4 – 8 (inversion-recovery).

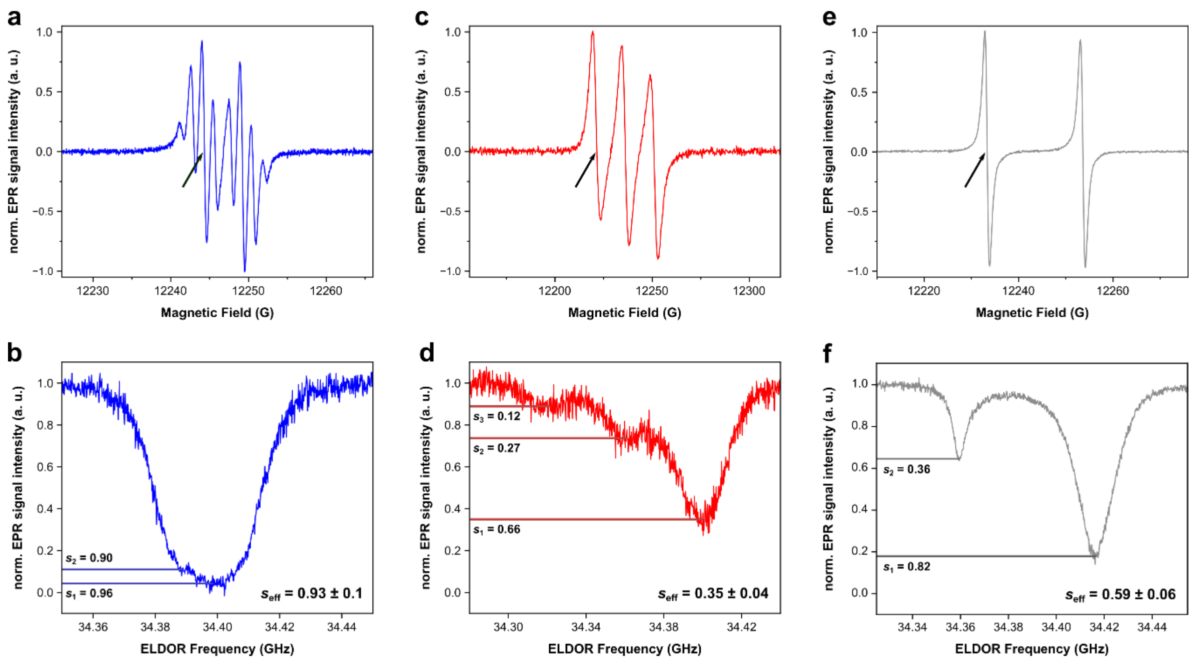

Figure S14: CW EPR (**a**, **c**, **e**) and ELDOR (**b**, **d**, **f**) spectra of galvinoxyl (blue), TEMPO (red), and TEMPONE-^{15}N-d_{16} (grey, 10 mM PA in neat hexafluorobenzene, for saturation factor estimation at 9.4 T, see Sec. S5) measured at 1.2 T using the same sample as for the experiments shown in Figure S13. The black arrow in the CW spectra indicate the detection field value for the ELDOR measurements. The individual saturation factors s_i were determined from the EPR signal intensity drops of the ELDOR spectra and the effective saturation factors were calculated. The error of the effective saturation factor is assumed to be 10%. EPR experimental parameters (galvinoxyl): ~34.4 GHz, 100 kHz modulation frequency, 0.2 G modulation amplitude, 60 dB receiver gain, 0.125 mW power, 16 scans, 2048 points and 50 G sweep width. EPR experimental parameters (TEMPO/TN): ~34.4 GHz, 100 kHz modulation frequency, 0.5 G modulation amplitude, 40 dB receiver gain, 0.125 mW power, 10/2 scans, 1024 points and 200 G/80 G sweep width. ELDOR experimental parameters: t_p(ELDOR) = 3 – 5 µs, $P_{MW} \approx 40$ W, t_p(90° det.) = 30 – 50 ns, VG = 27 – 42 dB, ns = 2 – 60, srt = 5 ms, spp = 10 – 50.

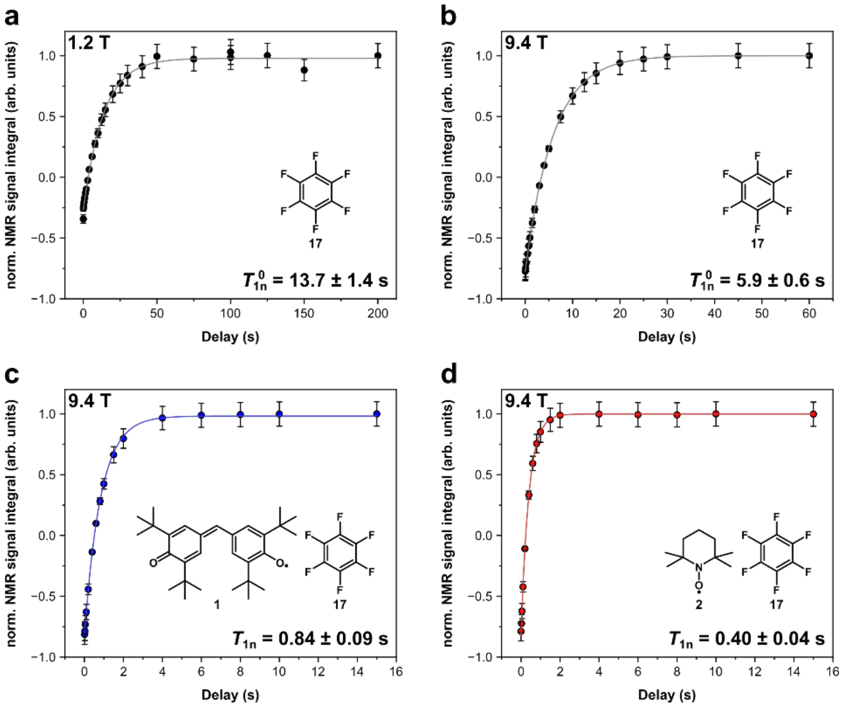

Figure S15: Plotted NMR signal integrals from inversion-recovery experiments and mono exponential fits (curves) to determine the ^{19}F nuclear relaxation times T_{1n} and T_{1n}^0 of hexafluorobenzene in presence and absence of the radical, respectively, at 1.2 T and 9.4 T. **a)** neat hexafluorobenzene (~8.7 M) without PA at 1.2 T, **b)** hexafluorobenzene (~500 mM) dissolved in CCl_4 without PA at 9.4 T, **c)** hexafluorobenzene (~500 mM) dissolved in CCl_4 with galvinoxyl (~10 mM) at 9.4 T, and **d)** hexafluorobenzene (~500 mM) dissolved in CCl_4 with TEMPO (~25 mM) at 9.4 T. The error of the NMR integrals and nuclear relaxation times are estimated to be 10%. ^{19}F NMR experimental parameters at 1.2 T: $T \approx 300$ K, $V_{sample} \approx 5$ µL, t_p(90° RF) = 3 – 3.5 µs, P_{RF} = 60 W, RD = 1 – 5 s, LB = 3 Hz, ns = 4 – 8. ^{19}F NMR experimental parameters at 9.4 T: $T \approx 300$ K, $V_{sample} \approx 20$ µL, t_p(90° RF) = 16.5 – 17 µs, P_{RF} = 21 W, RD = 5 – 15 s, LB = 3 Hz, ns = 4 – 8.

S5: Coupling factors from ^{19}F OE-DNP enhancements and simulation parameters

Many different ^{19}F DNP measurements of hexafluorobenzene doped with galvinoxyl or TEMPO are available in the literature at various magnetic field strengths. Values of the coupling factors are reported in Table S1. In cases where the coupling factor was not reported, we estimated the coupling factor based on the reported enhancement. Maximum and minimum leakage and saturation factors were estimated (if not reported) based on the radical concentration and the applied MW power during the DNP experiment. We acknowledge a significant uncertainty in estimating the saturation factor of experimental setups in the literature. To account for this, we assumed upper and lower boundaries for *s* (detailed in Table S1). These limits were used to calculate coupling factors and the error of ξ given in Table S1 reflects the lower and upper limits for *s*. A similar approach was used for our experimental data at 9.4 T, as electron spin relaxation parameters of galvinoxyl at 9.4 T were not available for the calculation of *s*. For the galvinoxyl/hexafluorobenzene system, we estimate, based on the ^{19}F enhancement and leakage factor, the lower limit of its coupling factor by assuming a saturation factor of $s < 1$ (see Tab. S1). To estimate the saturation factor of TEMPO in CCl_4 at 9.4 T, we assume the same field dependence of the the electron spin relaxation times like for TEMPONE,[3] due to similar chemical environment of the radical site. Because the Heisenberg exchange is field independent, and MW excitation is much narrower than the EPR linewidth, the saturation factor of TEMPO at 9.4 T can be calculated as:

$$s(\mathrm{TEMPO}, 9.4\ \mathrm{T}) = \frac{s(\mathrm{TEMPONE}, 9.4\ \mathrm{T})}{s(\mathrm{TEMPONE}, 1.2\ \mathrm{T})} \cdot s(\mathrm{TEMPO}, 1.2\ \mathrm{T}), \quad \text{eq. S1}$$

which gives 0.18 with 15% error (see Fig. S14). Our experimental values from the DNP measurements at 1.2 T and 9.4 T, as well as the data reported in the literature at different magnetic fields are summarized in Table S1. The symbols in Fig. 4a (main text) correspond to the data points from the last column in Table S1. Data shown in Tab. S1-S2 were partially reported in the PhD thesis of one author.[4]

To fit the field dependence of the coupling factors, we utilized dynamics models previously adopted to model ^{13}C coupling factors. We can express the coupling factor in form of transition probabilities:[7]

$$\xi = \frac{w_2^{\mathrm{dip}} - w_0^{\mathrm{dip}} - w_0^{\mathrm{sc}}}{\left(w_0^{\mathrm{dip}} + 2w_1^{\mathrm{dip}} + w_2^{\mathrm{dip}}\right) + w_0^{\mathrm{sc}}}, \quad \text{eq. S2}$$

where the subscript 'dip' denotes the dipolar relaxation terms and 'sc' the scalar relaxation terms. The terms w_1, w_0, and w_2, are the transition probabilities for single nuclear transitions, double and zero quantum transitions, respectively.

Equation S2 can also be expressed in terms of spectral densities:[8-10]

$$\xi = \frac{5}{7}\left(1 - \frac{3k_{\mathrm{D}}J_{\mathrm{D}}(\omega_{\mathrm{n}}, \tau_{\mathrm{D}}) + 3k_{\mathrm{rot}}J_{\mathrm{rot}}(\omega_{\mathrm{n}}, \tau_{\mathrm{C}})}{R_{1,\mathrm{D}} + R_{1,\mathrm{rot}} + R_{1,\mathrm{sc}}}\right) - \frac{12}{7}\left(\frac{R_{1,\mathrm{sc}}}{R_{1,\mathrm{D}} + R_{1,\mathrm{rot}} + R_{1,\mathrm{sc}}}\right) \quad \text{eq. S3.}$$

Here, k_D and k_{rot} are the prefactors of the translational (J_D) and rotational spectral density (J_{rot}), $R_{1,i}$ are the nuclear relaxation rates for translational diffusion (D), rotational diffusion (rot), and contact contributions (sc).

Table S1: Summary of determined and reported ^{19}F coupling factors of hexafluorobenzene using TEMPO or galvinoxyl as PA at different magnetic fields. Maximum and minimal coupling factors were calculated using the Overhauser equation (eq. 1), where the average then was used if no coupling factor was reported. For this case, the leakage and saturation factor were assumed and the error of the coupling factor covers the assumed range. Using our setups at 1.2 T and 9.4 T, the leakage factors were calculated (S4) and the saturation factors determined via ELDOR measurements (1.2 T, S4). All samples used neat hexafluorobenzene (~8.7 M) apart from TEMPO and galvinoxyl at 0.34 T and 3.4 T from ref.[11] where hexafluorobenzene was dissolved in benzene (1/4, v/v) and at 9.4 T, where we used ~500 mM dissolved in CCl_4.

**Radical**	**Field (T)**	c_{PA} **(mM)**	P_{MW} **(W)**	f	s	ε	$\lvert\xi^{max}_{min}\rvert$	ξ
TEMPO	0.34	20	5	0.85[a]<br>0.95[a]	0.4[a]<br>0.6[a]	−11.1[11]	0.051<br>0.030	0.041 ± 0.011
	0.34							0.094 ± 0.003[12]
	1.2*	10	40	0.96[b,c]	0.35[b,c]	2.2 ± 0.[b,d]		−0.005 ± 0.001[b,d]
	3.4	20	0.1	0.85[a]<br>0.95[a]	0.2[a]<br>0.3[a]	5.7[11]	−0.040[a]<br>−0.024[a]	−0.032 ± 0.008
	9.4*	25	50	0.93[b,c]	0.15[a]<br>0.21[a]	1.0 ± 0.1[b,c]	0.0001<br>0.00007	(9 ± 2) · 10^{-5}
Galvinoxyl	0.00153							0.287 ± 0.043[d,13]
	0.0074	10		1	1	−200 ± 20[e,14]		0.287 ± 0.043[c]
	0.0074	10		1	1	−145 ± 15[e,15]		0.209 ± 0.021[c]
	0.0175							0.250 ± 0.025[16]
	0.305	10		1	1	7.0 ± 0.7[e,15]		−0.009 ± 0.001[c]
	0.34	20	5	0.85[a]<br>0.95[a]	0.5[a]<br>0.7[a]	21.2[11]	−0.068<br>−0.043	−0.056 ± 0.013
	0.343							−0.050 ± 0.005[16]
	0.365	10		1	1	13.0 ± 1.3[e,15]		−0.017 ± 0.002[c]
	0.89	10		1	1	29.0 ± 2.9[e,15]		−0.040 ± 0.004[c]
	1.2	25	3	0.85[a]<br>0.95[a]	0.3[a]<br>0.6[a]	13.5[11]	−0.070<br>−0.031	−0.051 ± 0.020
	1.2*	10	40	0.80[b,c]	0.93[b,c]	37 ± 4[b,c]		−0.070 ± 0.011[b,d]
	1.323							−0.080 ± 0.008[16]
	3.4	20	0.1	0.85[a]<br>0.95[a]	0.2[a]<br>0.4[a]	20.1[11]	−0.161<br>−0.072	−0.116 ± 0.045
	9.4*	10	50	0.86[b,c]	<1[a,f]	16 ± 2[b,d]		<−0.025

*This work. [a]Value was estimated. [b]Value was obtained experimentally. [c]Error is assumed to be 10%. [d]Error is assumed to be 15%. [e]Enhancements reported at infinite power with f = 1 and s = 1. [f]For estimation of the lower limit of the coupling factor.

Translational diffusion of the two molecules relative to each other is described by the force-free-hard-sphere model introduced by Freed and co-workers.[17, 18] The spectral density is given by:

$$J_{\mathrm{D}}(\omega_{\mathrm{i}},\tau_{\mathrm{D}}) = \frac{1+\frac{5z}{8}+\frac{z^2}{8}}{1+z+\frac{z^2}{2}+\frac{z^3}{6}+\frac{4z^4}{81}+\frac{z^5}{81}+\frac{z^6}{648}} \quad \text{eq. S4,}$$

with $z=\sqrt{2\omega_i\tau_{\mathrm{D}}}$ and ω_i being the electron (e) or nuclear (n) larmor frequency, respectively. Here, τ_{D} is the translational correlation time $\tau_{\mathrm{D}}=\frac{r_{\mathrm{D}}^2}{D_{\mathrm{T}}+D_{\mathrm{PA}}}$, with r_{D}being the distance of closest approach and D_{i} the diffusion constant of the target and the PA, respectively. For the simulations we set $D_{\mathrm{T}}+D_{\mathrm{PA}}=D_{\mathrm{T}}$. The translational prefactor is given by:

$$k_{\mathrm{D}} = \frac{32000\pi}{405}\left(\frac{\mu_0}{4\pi}\right)^2 \frac{N_{\mathrm{A}}c\gamma_{\mathrm{n}}^2 g_{\mathrm{e}}^2\mu_{\mathrm{B}}^2 S(S+1)}{r_{\mathrm{D}}(D_{\mathrm{T}}+D_{\mathrm{PA}})} \quad \text{eq. S5.}$$

Here, N_A is the Avogadro constant, c the concentration of the PA, γ_n the nuclear gyromagnetic ratio, g_e the electron g-factor, S is the electron spin quantum number, μ_0 the permeability constant and μ_B the Bohr magneton. The translational diffusion relaxation rate is then given by:

$$R_{1,D} = k_D[7J_D(\omega_e, \tau_D) + 3J_D(\omega_n, \tau_D)] \quad \text{eq. S6.}$$

The rotational spectral density is given by:[9]

$$J_{rot}(\omega_i, \tau_D) = \frac{\tau_C}{1 + \omega_i^2 \tau_C^2} \quad \text{eq. S7}$$

with the rotational correlation time τ_C and the corresponding relaxation rate $R_{1,rot}$:

$$R_{1,rot} = k_{rot}[7J_{rot}(\omega_e, \tau_C) + 3J_{rot}(\omega_n, \tau_C)] \quad \text{eq. S8.}$$

The prefactor k_{rot} is used as a scaling factor. Lastly, the spectral density of the contact interaction is given by:[19, 20]

$$J_{sc}(\omega_e, \tau_{sc,i}) = \sum_{i=1}^{n} F_i[\tau_{sc,i} \cdot \exp\{-\tau_{sc,i}\omega_e\}]^2 \quad \text{eq. S9}$$

that can include different types of collisions i. Here, $F_i = \frac{\langle A_i^2 \rangle}{\hbar^2 \tau_{p,i}}$ is with the isotropic hyperfine coupling A_i, $\tau_{p,i}$ the time between two collisions of same type, $\hbar$ the reduced Planck constant and $2\tau_{sc,i}$ is the collision duration. The corresponding relaxation rate is:[10]

$$R_{1,sc} = \frac{2}{3} S(S+1) J_{sc}(\omega_e, \tau_{sc,i}) \quad \text{eq. S10.}$$

Table S2: Amplitudes and correlation times that were employed as best-fit parameters for modeling the ^{19}F coupling factors as a function of the magnetic field using eq. S3 with TEMPO or galvinoxyl as polarizing agent (Fig. 4a). Translational correlation times are similar to the values reported in ref.[12] (TEMPO) and ref.[16] (galvinoxyl) and the distances of closest approach r_D were calculated using the self diffusion coefficient $D_T = 1.6 \cdot 10^{-9}$ m²/s of hexafluorobenzene at 25 °C[21] as $r_D = \sqrt{\tau_D \cdot D_T}$. The order of magnitude of the rotational correlation time is comparable to estimations by EPR measurements (Fig. S19) and to the reported value of galvinoxyl in toluene.[22] The concentration c was set to 0.02 M for both radicals and $F_i = \langle A_i^2 \rangle / (\hbar^2 \tau_{p,i})$ is used. All other parameters were varied for the best-fit of the coupling factor values. The error of the fitting parameters are assumed to be 20% accounting for the reliability of the experimental data.

Radical	Translation		Rotation		Contact 1		Contact 2	
	r_D (Å)	τ_D (ps)	k_{rot} (s^{-2})	τ_C (ps)	F_1 (rad²/s²)	$\tau_{sc,1}$ (ps)	F_2 (rad²/s²)	$\tau_{sc,2}$ (ps)
TEMPO	2.8	50			$1.69 \cdot 10^{24}$	1.0		
Galvinoxyl	2.75	50	$10 \cdot 10^9$	40	$2.5 \cdot 10^{23}$	4.1	$1.68 \cdot 10^{25}$	0.3

S6: Quantum chemical calculations

Optimal geometries of the PA and target molecule complex and isotropic hyperfine couplings were obtained with density functional theory (DFT) calculations. To compute the conformational space around the polarizing agent and the target molecule, the CREST program (Conformer-Rotamer Ensemble Sampling Tool, ver. 2.12) was employed.[23] For galvinoxyl/C_6F_6 and TEMPO/C_6F_6 the nci-flag was used (non-covalent interaction), while it was omitted for galvinoxyl/fluorobenzene system to avoid output of identical conformers. In total 36, 31, and 131 complexes were identified for galvinoxyl/C_6F_6, TEMPO/C_6F_6 and galvinoxyl/fluorobenzene, respectively. The input for CREST consisted of the pre-optimized monomer geometries obtained with ORCA.

Geometry optimization of each conformer was computed using ORCA (ver. 5.0.4)[24, 25] B3LYP was employed as functional with the def2-TZVPP basis set.[26-30] The auxiliary basis set def2/J[31] and resolution of identity for coulomb integrals and chains of spheres exchange (RIJCOSX)[32, 33] were used as well as Grimmes' atom-pairwise dispersion correction with Becke-Johnson damping scheme (D3BJ)[34, 35] to consider dispersion interactions. TIGHTOPT optimization procedure and very tight self-consistent field convergence criteria (VERYTIGHTSCF) were applied. The implicit solvent model conductor-like polarization continuum model (C-PCM) for CCl_4 was included to mimic the effect of a solvent.[36] Default gridsize was used for all calculations. In the last step, the isotropic hyperfine couplings A_{iso} to ^{19}F of the optimized radical-target complexes were computed using the EPR-III basis set.[37] The PyMOL Molecular Graphics System program (ver. 2.5.2) was used for the complex geometries displayed in the figures. In Fig. S16 and S17 all optimized geometries are displayed superimposed showing the possible arrangements of the target molecule with respect to the polarizing agent. Fig. 4d shows a plot of the isotropic hf couplings as function of the relative energies of the complexes for the two systems. Most optimized PA/target complexes were not symmetrical and yielded different hfc values for each fluorine nucleus. Data shown in Fig. S16-S18 were initially reported in the PhD thesis of one author.[4]

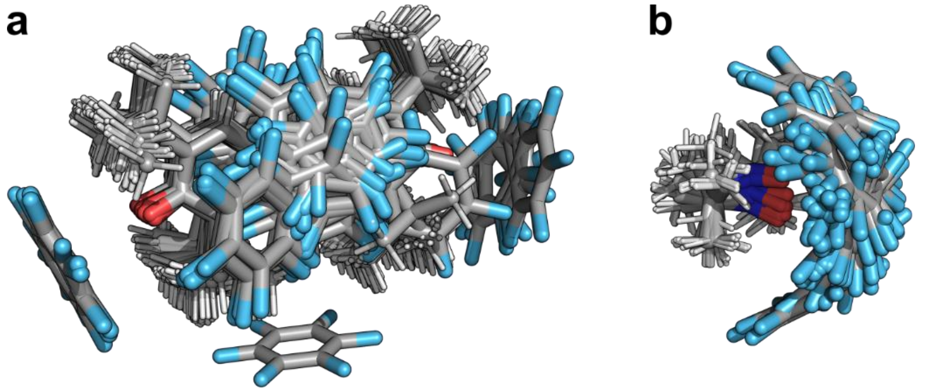

Figure S16: All optimized and superimposed complex geometries of the two system galvinoxyl/C_6F_6 and TEMPO/C_6F_6 showing the possible arrangements of the target molecule with respect to the radical. **a)** 36 superimposed complexes of galvinoxyl and hexafluorobenzene. **b)** 31 superimposed complexes of TEMPO and hexafluorobenzene. The number of complexes is a result of the conformer search by CREST. The structures were further optimized using ORCA at higher level of theory. ORCA 5.0.4 input line: B3LYP def2-TZVPP CPCM(ccl4) def2/J RIJCOSX D3BJ TIGHTOPT VERYTIGHTSCF. These geometries were used to calculate the isotropic hyperfine couplings to the ^{19}F nuclei with B3LYP EPR-III CPCM(ccl4) def2/J RIJCOSX D3BJ VERYTIGHTSCF.

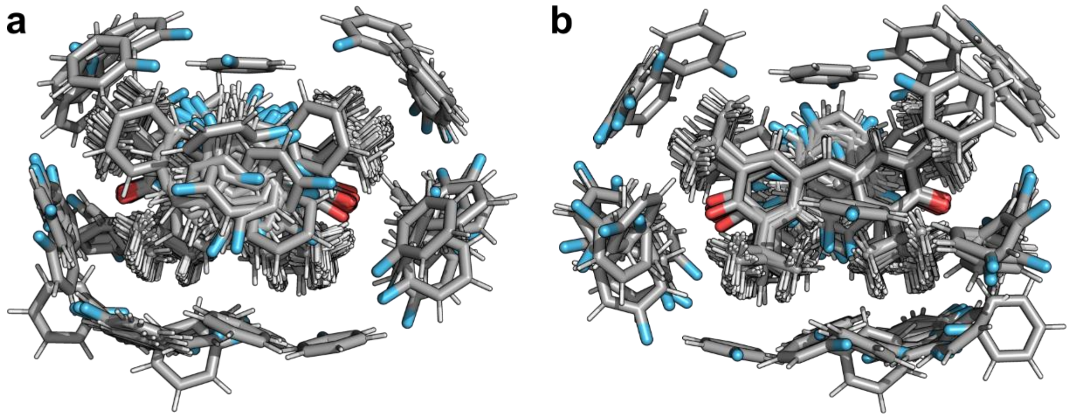

Figure S17: Front view (**a**) and back view (**b**) of all superimposed optimized complex geometries of the system galvinoxyl and fluorobenzene (131 total). The number of complexes is a result of the conformer search CREST. The structures were further optimized using ORCA at higher level of theory. ORCA 5.0.4 input line: B3LYP def2-TZVPP CPCM(ccl4) def2/J RIJCOSX D3BJ TIGHTOPT VERYTIGHTSCF. These complexes were used to calculate the isotropic hyperfine couplings to the ^{19}F and ^{1}H nuclei with B3LYP EPR-III CPCM(ccl4) def2/J RIJCOSX D3BJ VERYTIGHTSCF.

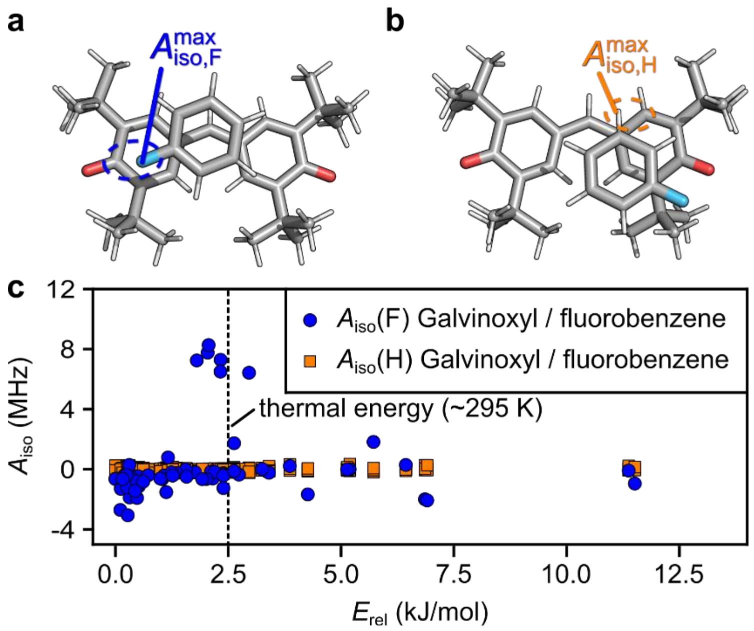

Figure S18: **a**, **b)** DFT optimized structures with maximum ^{19}F A_{iso} of galvinoxyl/fluorobenzene (**a**, A_{iso}(F) ≈ 8.3 MHz, E_{rel} ≈ 2.1 kJ/mol) and maximum ^{1}H A_{iso} of galvinoxyl/fluorobenzene (**b**, A_{iso}(F) ≈ 0.35 MHz, E_{rel} ≈ 5.2 kJ/mol)**. c)** Hyperfine coupling A_{iso} of ^{19}F (blue) and ^{1}H (orange) of for each optimized galvinoxyl/fluorobenzene structure plotted against the relative single-point energy of the complex.

S7: EPR characterization of galvinoxyl radical at 9.4 Tesla

263 GHz EPR was performed at room temperature (RT) and at 40 K to characterize the EPR spectrum of the galvinoxyl radical.263 GHz CW- and pulse EPR experiments were performed on a Bruker ElexSys E780 EPR spectrometer. All experiments were performed using a cylindrical TE_{012}-mode EPR/ENDOR resonator (Bruker BioSpin, model E9501510). Samples were loaded into quartz capillaries, I.D. = 0.2 mm, O.D. = 0.33 mm, and measured in volumes of around 50 nL.

For RT measurements, a stock sample solution (~100 µL) was degassed by 5 freeze-pump-thaw cycles and transferred to a glove box. Then, the quartz capillaries were filled with the solution under N_2 atmosphere and both ends were closed with sealing rubber (Critoseal®). The filled capillaries were transported to the spectrometer under N_2 atmosphere and only upon insertion into the resonator were exposed to air. The measurements were performed directly after inserting the samples into the resonator.

No degassing was applied for low-temperature ESE. The samples were loaded into the capillaries, inserted into the resonator and quickly frozen by immersing the loaded resonator in liquid nitrogen. The cold resonator was then transported into the precooled (80 K) sample cryostat and the temperature was stabilized at 40 K.

From the ESE spectrum in the frozen state, we determined following *g* values for the galvinoxyl radical: $g_x = 2.00647$, $g_y = 2.00436$, $g_z = 2.00231$ (error ± 0.00003) using BDPA-d_{27} radical as a reference standard (Figure S19a). This leads to an isotropic *g* value of $g_{iso} = 2.00438$, consistent with the CW EPR measurement at room temperature.

Experimental parameters for CW-EPR: MW frequency - 263.185 GHz, MW power – 1.6 mW, modulation frequency – 100 kHz, modulation amplitude - 0.5 G, receiver gain – 39 dB, number of scans - 5, conversion time – 82 ms, room temperature.

Experimental parameters for ESE: MW frequency - 263.185 GHz, MW power – 50 mW, $\pi/2$-τ-π Hahn echo sequence with $\pi/2 = 32$ ns, $\pi = 64$ ns and $\tau = 300$ ns, repetition time – 50 ms, shots per point -10, number of scans - 19 and 32 scans for Galvinoxyl with deuterated BDPA and Galvinoxyl only, temperature – 40 K.

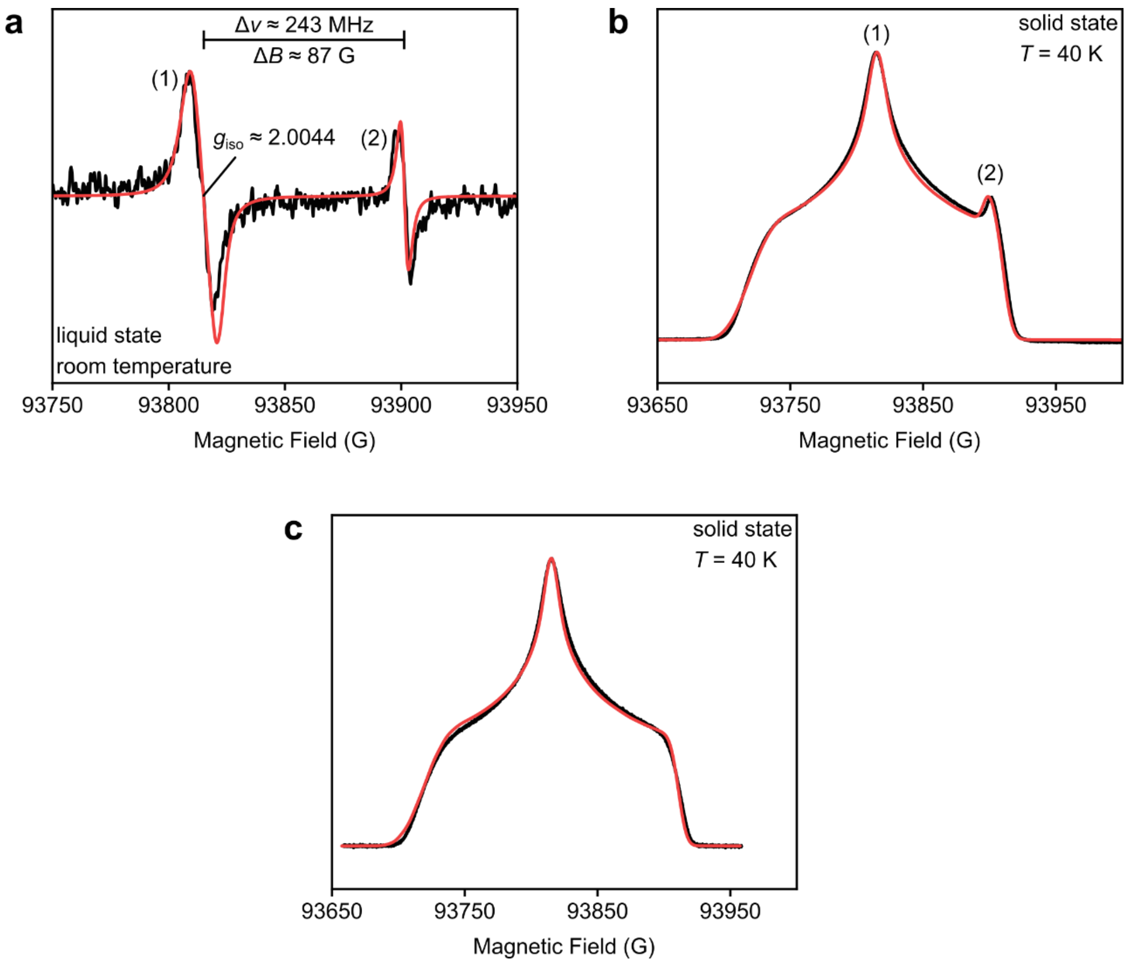

Figure S19: **a)** Experimental (black)[3] and simulated (red) 263 GHz CW EPR spectrum of (1) Galvinoxyl (10 mM) and (2) deuterated BDPA (10 mM) in toluene measured at room temperature. **b)** Experimental (black) and theoretical (red) 263 GHz echo detected EPR (ESE) spectrum of (1) Galvinoxyl (10 mM) and (2) deuterated BDPA (10 mM) in Toluene measured at 40 K. **c)** Experimental (black) and simulated (red) 263 GHz ESE spectrum of 10 mM Galvinoxyl in Toluene measured at 40 K. Simulation parameters are: Galvinoxyl - g_x = 2.00647, g_y = 2.00436, g_z = 2.00231; BDPA - g_x = 2.00263, g_y = 2.00259, g_z = 2.00234, Hyperfine galvinoxyl: hfc($^{1}H_{Methylen}$) = [40.31 0.46 6.0] MHz + 4 protons with hfc(^{1}H) = [25.78 1.45 −7.64] MHz, Hyperfine BDPA: hfc(^{2}H) = [−0.9 −1.25 −0.3] MHz, hfc(^{2}H) = [0.3 0.15 0.25] MHz, correlation time for the liquid-state simulation: Galvinoxyl = 0.086 ns, BDPA = 10.0 ns.

S8: References

(1) Xiao, J.; Wong, Z. Z.; Lu, Y. P.; Loh, T. P. Hexahydropyrrolo[2,3-b]indoles: A New Class of Structurally Rigid Tricyclic Skeleton for Oxazaborolidine-Catalyzed Asymmetric Borane Reduction. *Adv. Synth. Catal.* **2010**, *352* (7), 1107-1112. DOI: 10.1002/adsc.200900908.

(2) Deboves, H. J. C.; Montalbetti, C. A. G. N.; Jackson, R. F. W. Direct synthesis of Fmoc-protected amino acids using organozinc chemistry: application to polymethoxylated phenylalanines and 4-oxoamino acids. *J. Chem. Soc., Faraday Trans.* **2001**, (16), 1876-1884. DOI: 10.1039/b103832j.

(3) Levien, M.; Yang, L.; van der Ham, A.; Reinhard, M.; John, M.; Purea, A.; Ganz, J.; Marquardsen, T.; Tkach, I.; Orlando, T.; et al. Overhauser enhanced liquid state nuclear magnetic resonance spectroscopy in one and two dimensions. *Nat. Commun.* **2024**, *15* (1). DOI: 10.1038/s41467-024-50265-5.

(4) Reinhard, M. Development of ^{19}F and ^{31}P Dynamic Nuclear Polarization in the Liquid State. PhD Thesis, Göttingen, 2024.

(5) Orlando, T.; Dervişoğlu, R.; Levien, M.; Tkach, I.; Prisner, T. F.; Andreas, L. B.; Denysenkov, V. P.; Bennati, M. Dynamic Nuclear Polarization of ^{13}C Nuclei in the Liquid State over a 10 Tesla Field Range. *Angew. Chem, Int. Ed.* **2018**, *58* (5), 1402-1406. DOI: 10.1002/anie.201811892.

(6) Türke, M.-T.; Bennati, M. Saturation factor of nitroxide radicals in liquid DNP by pulsed ELDOR experiments. *Phys. Chem. Chem. Phys.* **2011**, *13* (9), 3630-3633. DOI: 10.1039/c0cp02126a.

(7) Hausser, K. H.; Stehlik, D. Dynamic Nuclear Polarization in liquids. *Adv. Magn. Reson.* **1968**, *3*, 79-139. DOI: 10.1016/B978-1-4832-3116-7.50010-2.

(8) Parigi, G.; Ravera, E.; Bennati, M.; Luchinat, C. Understanding Overhauser Dynamic Nuclear Polarisation through NMR relaxometry. *Mol. Phys.* **2019**, *117* (7–8), 888–897. DOI: 10.1080/00268976.2018.1527409.

(9) Ravera, E.; Luchinat, C.; Parigi, G. Basic facts and perspectives of Overhauser DNP NMR. *J. Magn. Reson.* **2016**, *264*, 78-87. DOI: 10.1016/j.jmr.2015.12.013.

(10) Bennati, M.; Luchinat, C.; Parigi, G.; Türke, M.-T. Water ^{1}H relaxation dispersion analysis on a nitroxide radical provides information on the maximal signal enhancement in Overhauser dynamic nuclear polarization experiments. *Phys. Chem. Chem. Phys.* **2010**, *12* (22). DOI: 10.1039/c002304n.

(11) George, C.; Chandrakumar, N. Chemical-Shift-Resolved ^{19}F NMR Spectroscopy between 13.5 and 135 MHz: Overhauser–DNP-Enhanced Diagonal Suppressed Correlation Spectroscopy. *Angew. Chem, Int. Ed.* **2014**, *126* (32), 8581-8584. DOI: 10.1002/ange.201402320.

(12) Neudert, O.; Mattea, C.; Spiess, H. W.; Stapf, S.; Münnemann, K. A comparative study of ^{1}H and ^{19}F Overhauser DNP in fluorinated benzenes. *Phys. Chem. Chem. Phys.* **2013**, *15* (47), 20717-20726. DOI: 10.1039/c3cp52912f.

(13) Peksoz, A.; Yalciner, A.; Cimenoglu, M. A. A Low Field Fluorine-Electron Double Resonance Study for GALV and BDPA in Some Aliphatic and Aromatic Solvents. *Z. Naturforsch.* **2009**, *64a*, 477 – 484 DOI: 10.1515/zna-2009-7-810.

(14) Poindexter, E. H.; Stewart, J. R.; Caplan, P. J. Dynamic Polarization of Fluorine Nuclei in Solutions of Selected Free Radicals. *J. Chem. Phys.* **1967**, *47* (8), 2862-2873. DOI: 10.1063/1.1712309.

(15) Webb, R. H.; Nghia, N. v.; Pearlman, M. R.; Poindexter, E. H.; Caplan, P. J.; Potenza, J. A. Dynamic Nuclear Polarization: Collision Mechanics in Fluorocarbon Solutions. *J. Chem. Phys.* **1969**, *50* (10), 4408-4417. DOI: 10.1063/1.1670911.

(16) Müller-Warmuth, W.; Van Steenwinkel, R.; Noack, F. Dynamic Nuclear Polarization Experiments on ^{19}F in Solutions and their Interpretation by the "Pulse Model" of Molecular Collisions. *Z. Naturforsch.* **1968**, *23a*, 506-513. DOI: 10.1515/zna-1968-0408.

(17) Polnaszek, C. F.; Bryant, R. G. Nitroxide radical induced solvent proton relaxation: Measurement of localized translational diffusion. *J. Chem. Phys.* **1984**, *81* (9), 4038-4045.

(18) Hwang, L.-P.; Freed, J. H. Dynamic effects of pair correlation functions on spin relaxation by translational diffusion in liquids. *J. Chem. Phys.* **1975**, *63* (9), 4017-4025. DOI: 10.1063/1.431841.

(19) Müller-Warmuth, W.; Vilhjalmsson, R.; Gerlof, P. A. M.; Smidt, J.; Trommel, J. Intermolecular interactions of benzene and carbon tetrachloride with selected free radicals in solution as studied by ^{13}C and ^{1}H dynamic nuclear polarization. *Mol. Phys.* **1976**, *31* (4), 1055-1067. DOI: 10.1080/00268977600100811.
(20) Noack, F.; Krüger, G. J.; Müller-Warmuth, W.; Van Steenwinkel, R. Stochastische Prozesse in Spinsystemen. *Z. Naturforsch.* **1967**, *22a*, 2102-2108.
(21) Hogenboom, D. L.; Krynicki, K.; Sawyer, D. W. Self-diffusion and density of liquid hexafluorobenzene as a function of pressure and temperature. *Mol. Phys.* **2006**, *40* (4), 823-835. DOI: 10.1080/00268978000101911.
(22) Meyer, V.; Eaton, S. S.; Eaton, G. R. X-band Electron Spin Relaxation Times for Four Aromatic Radicals in Fluid Solution and Comparison with Other Organic Radicals. *Appl. Magn. Reson.* **2014**, *45* (10), 993-1007. DOI: 10.1007/s00723-014-0579-6.
(23) Pracht, P.; Bohle, F.; Grimme, S. Automated exploration of the low-energy chemical space with fast quantum chemical methods. *Phys. Chem. Chem. Phys.* **2020**, *22* (14), 7169-7192. DOI: 10.1039/c9cp06869d.
(24) Neese, F. The ORCA program system. *WIREs Computational Molecular Science* **2012**, *2* (1), 73-78. DOI: 10.1002/wcms.81.
(25) Neese, F. Software update: The ORCA program system—Version 5.0. *WIREs Computational Molecular Science* **2022**, *12* (5), e1606. DOI: 10.1002/wcms.1606.
(26) Weigend, F.; Ahlrichs, R. Balanced basis sets of split valence, triple zeta valence and quadruple zeta valence quality for H to Rn: Design and assessment of accuracy. *Phys. Chem. Chem. Phys.* **2005**, *7* (18). DOI: 10.1039/b508541a.
(27) Stephens, P. J.; Devlin, F. J.; Chabalowski, C. F.; Frisch, M. J. Ab Initio Calculation of Vibrational Absorption and Circular Dichroism Spectra Using Density Functional Force Fields. *J. Phys. Chem.* **2002**, *98* (45), 11623-11627. DOI: 10.1021/j100096a001.
(28) Becke, A. D. Density-functional thermochemistry. III. The role of exact exchange. *J. Chem. Phys.* **1993**, *98* (7), 5648-5652. DOI: 10.1063/1.464913.
(29) Lee, C.; Yang, W.; Parr, R. G. Development of the Colle-Salvetti correlation-energy formula into a functional of the electron density. *Phys. Rev. B* **1988**, *37* (2), 785-789. DOI: 10.1103/PhysRevB.37.785.
(30) Vosko, S. H.; Wilk, L.; Nusair, M. Accurate spin-dependent electron liquid correlation energies for local spin density calculations: a critical analysis. *Can. J. Phys.* **1980**, *58* (8), 1200-1211. DOI: 10.1139/p80-159.
(31) Weigend, F. Accurate Coulomb-fitting basis sets for H to Rn. *Phys. Chem. Chem. Phys.* **2006**, *8* (9). DOI: 10.1039/b515623h.
(32) Neese, F.; Wennmohs, F.; Hansen, A.; Becker, U. Efficient, approximate and parallel Hartree–Fock and hybrid DFT calculations. A 'chain-of-spheres' algorithm for the Hartree–Fock exchange. *Chem. Phys.* **2009**, *356* (1-3), 98-109. DOI: 10.1016/j.chemphys.2008.10.036.
(33) Neese, F. An improvement of the resolution of the identity approximation for the formation of the Coulomb matrix. *J. Comput. Chem* **2003**, *24* (14), 1740-1747. DOI: 10.1002/jcc.10318.
(34) Grimme, S.; Ehrlich, S.; Goerigk, L. Effect of the damping function in dispersion corrected density functional theory. *J. Comput. Chem* **2011**, *32* (7), 1456-1465. DOI: 10.1002/jcc.21759.
(35) Grimme, S.; Antony, J.; Ehrlich, S.; Krieg, H. A consistent and accurateab initioparametrization of density functional dispersion correction (DFT-D) for the 94 elements H-Pu. *J. Chem. Phys.* **2010**, *132* (15). DOI: 10.1063/1.3382344.
(36) Barone, V.; Cossi, M. Quantum Calculation of Molecular Energies and Energy Gradients in Solution by a Conductor Solvent Model. *J. Phys. Chem. A* **1998**, *102* (11), 1995-2001. DOI: 10.1021/jp9716997.
(37) Barone, V. Structure, Magnetic Properties and Reactivities of Open-Shell Species From Density Functional and Self-Consistent Hybrid Methods. *Recent Advances in Density Functional Methods* **1995**, *1*. DOI: 10.1142/9789812830586_0008.
